# Supplementary material for: Preparation of Chiral Enantioenriched Densely Substituted Cyclopropyl Azoles, Amines, and Ethers via Formal SN2′ Substitution of Bromocylopropanes
Source: Molecules. 2022 Oct 20;27(20):7069. doi: 10.3390/molecules27207069 (PMC9609026; doi:10.3390/molecules27207069)
Supplement: Supplementary file 1 [file molecules-27-07069-s001.zip › molecules-1960386-supplementary.pdf]

Supplemental Information

Preparation of Chiral Enantioenriched Densely Substituted Cyclopropyl Azoles, Amines, and Ethers via Formal SN2' Substitution of Bromocylopropanes

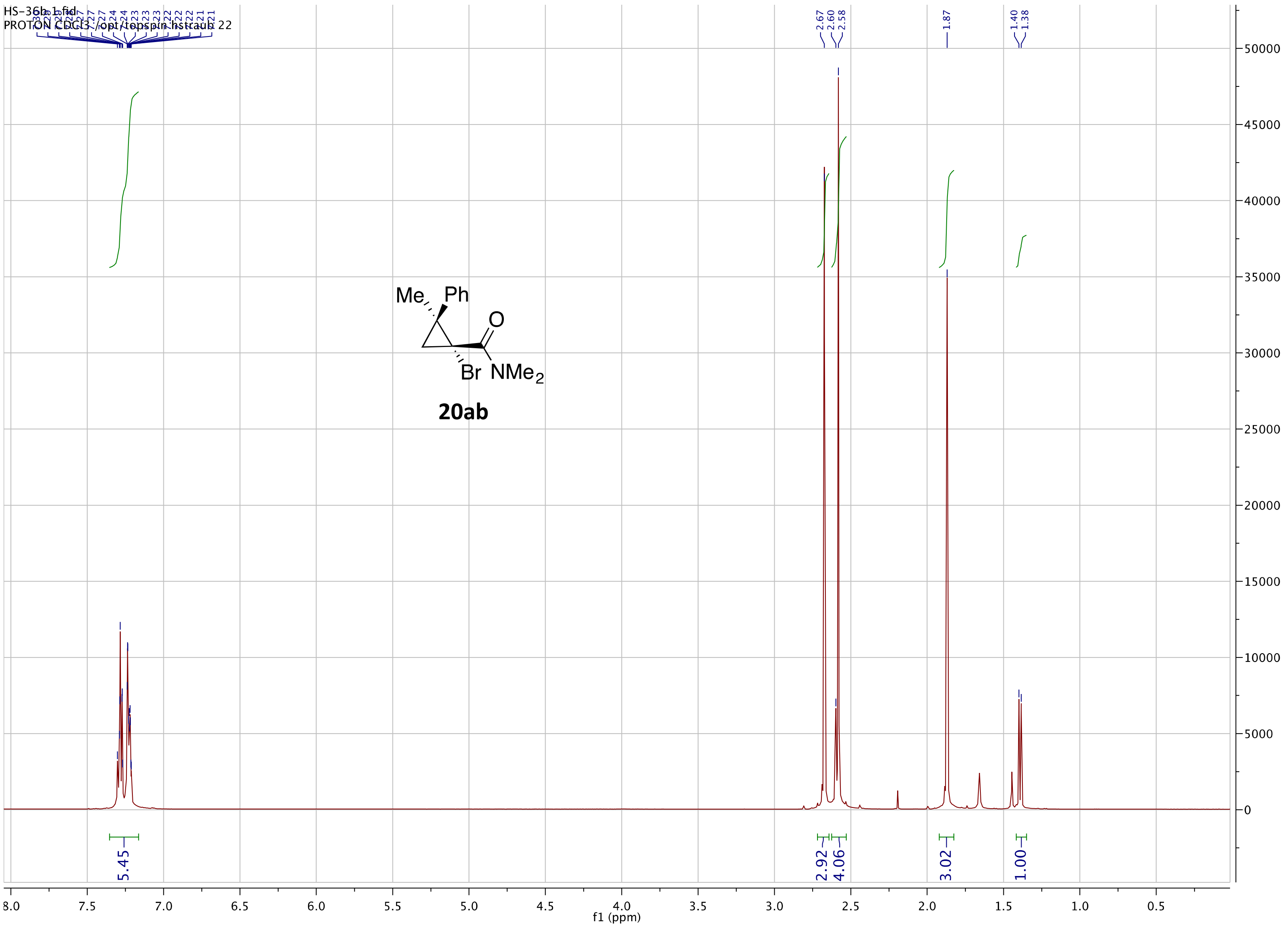

Figure S1. ^{1}H NMR spectrum of compound **20ab.**

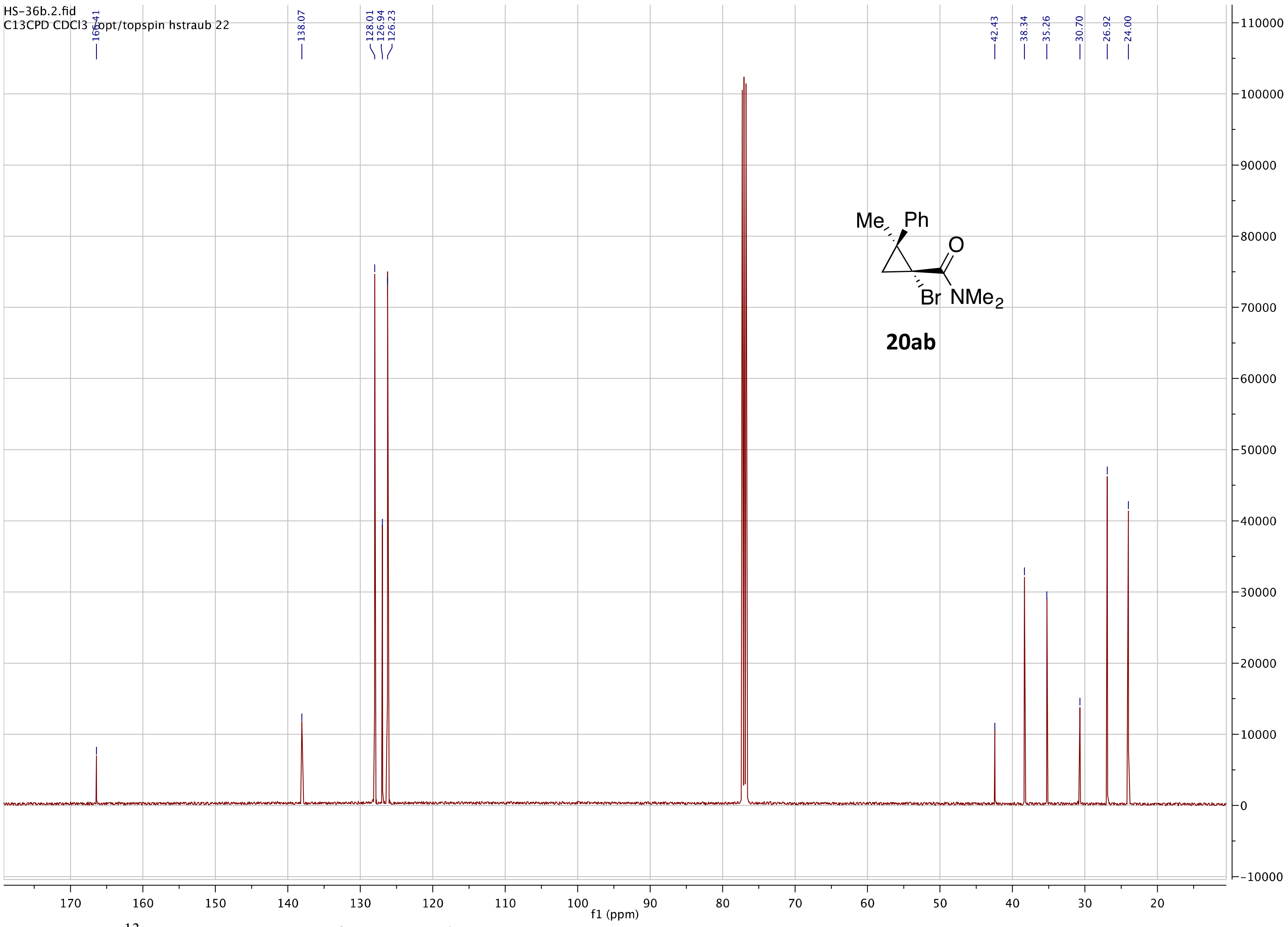

Figure S2. ^{13}C NMR spectrum of compound **20ab.**

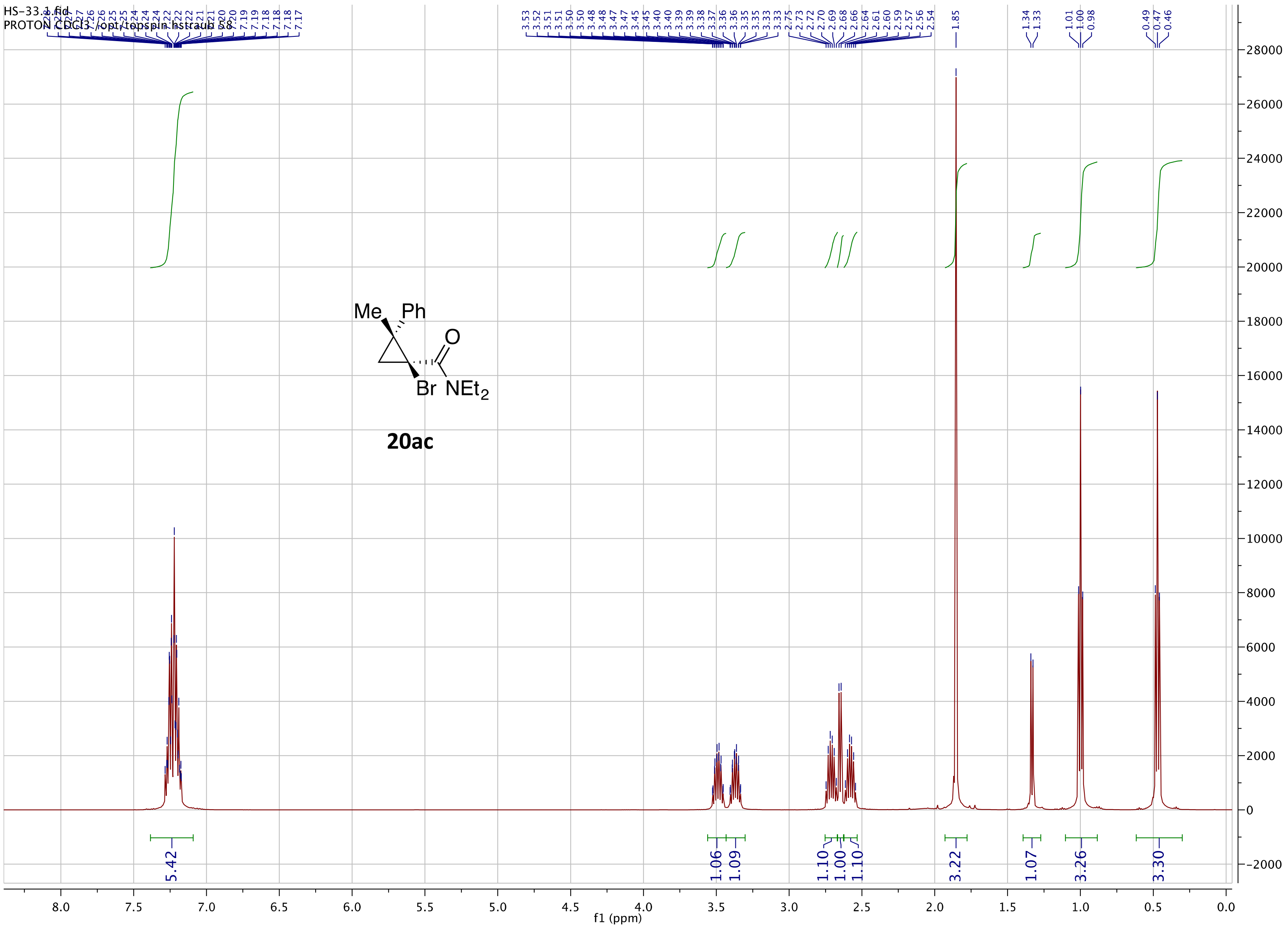

Figure S3. ^{1}H NMR spectrum of compound **20ac**

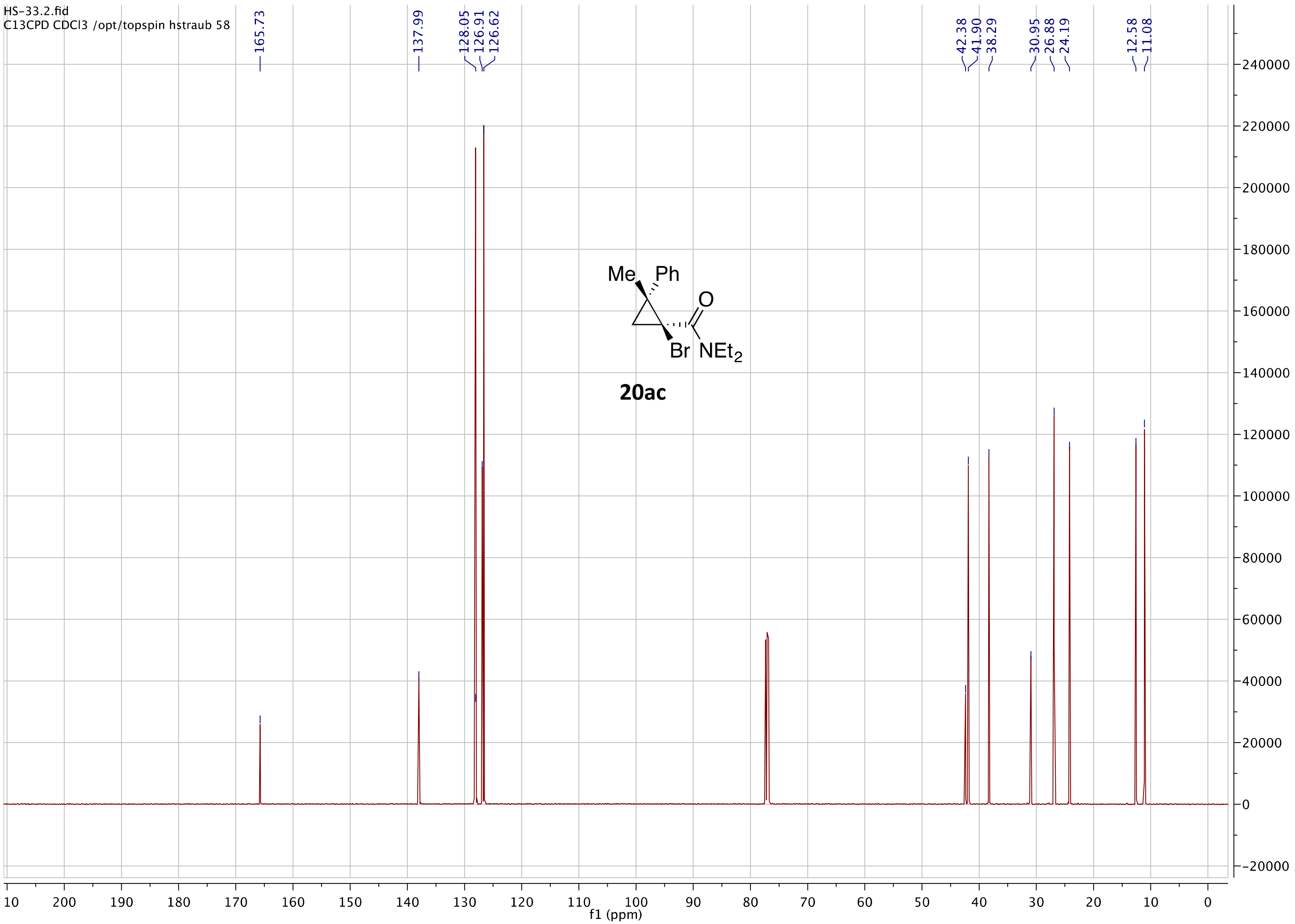

Figure S4. ^{13}C NMR spectrum of compound **20ac**

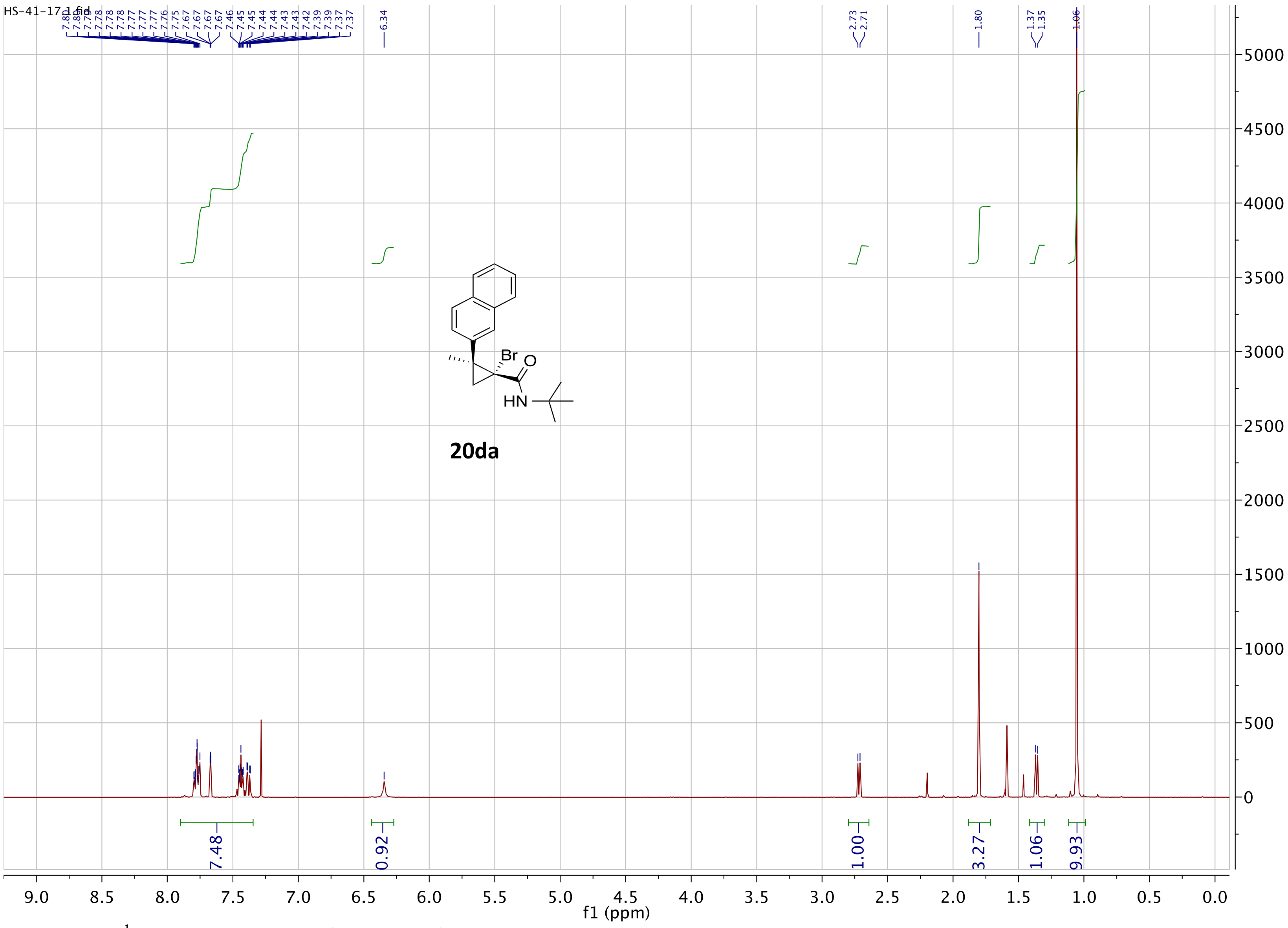

Figure S5. ^{1}H NMR spectrum of compound **20da**

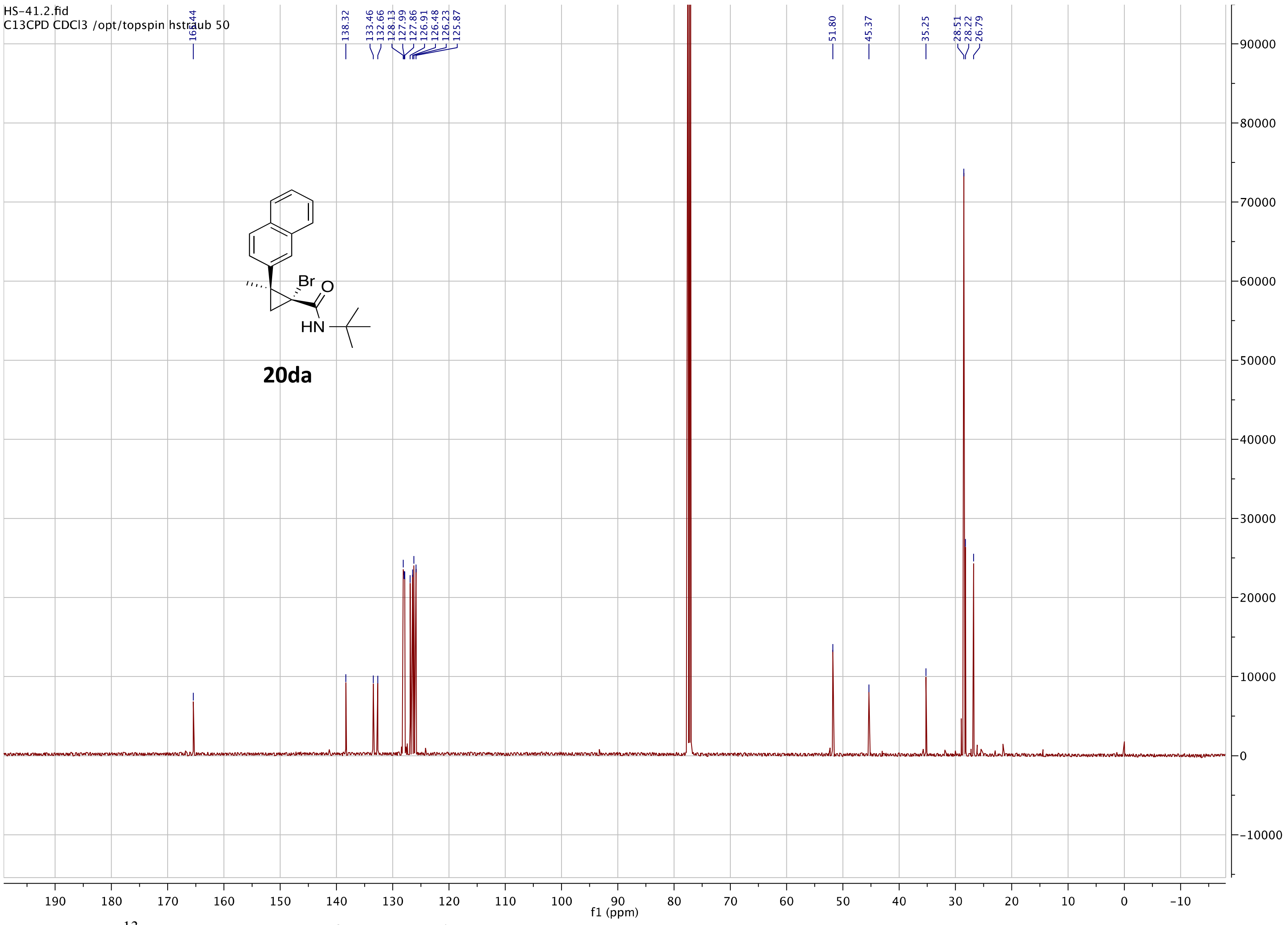

Figure S6. ^{13}C NMR spectrum of compound **20da**

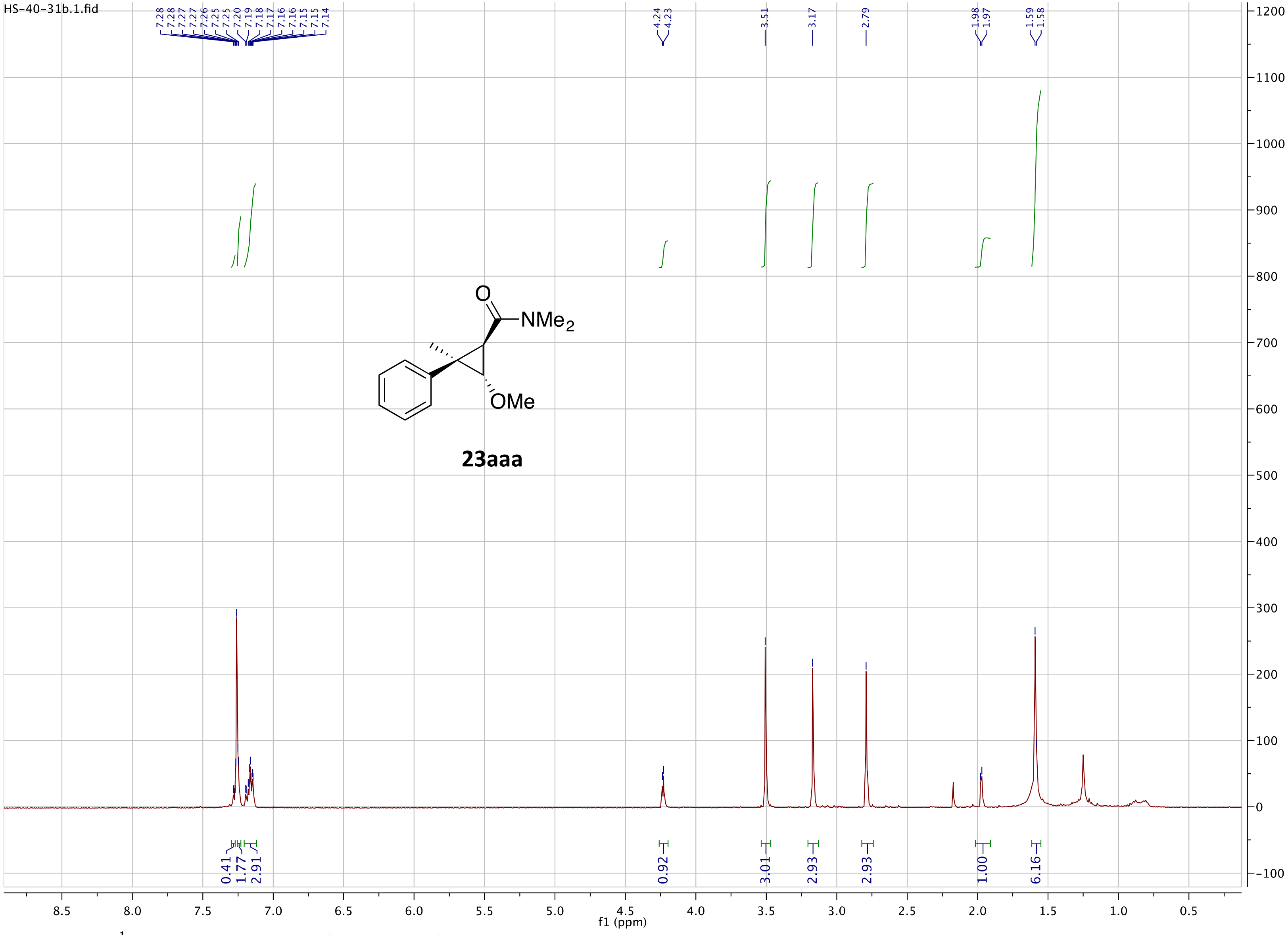

Figure S7. ^{1}H NMR spectrum of compound **23aaa**

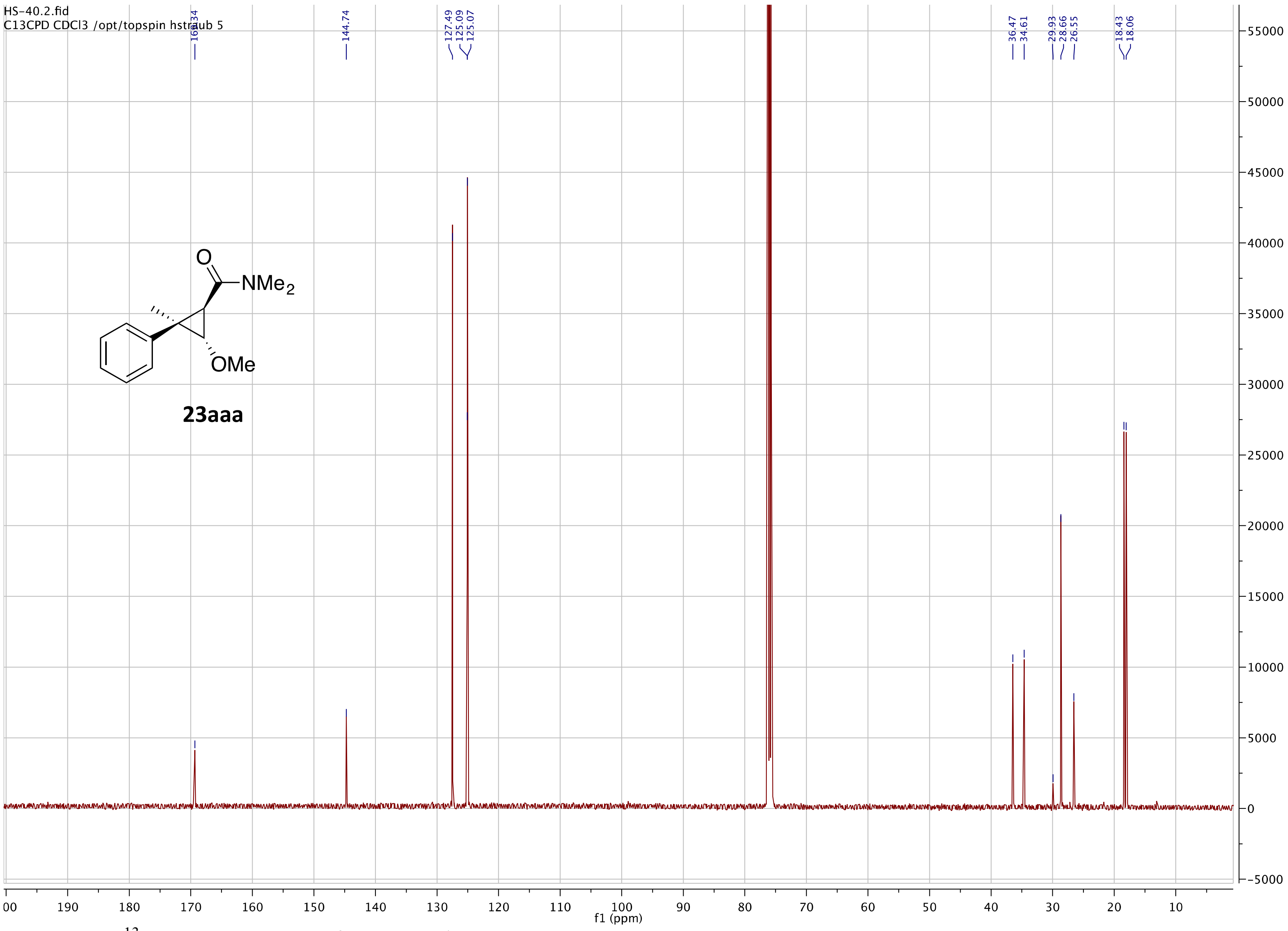

Figure S8. ^{13}C NMR spectrum of compound **23aaa**

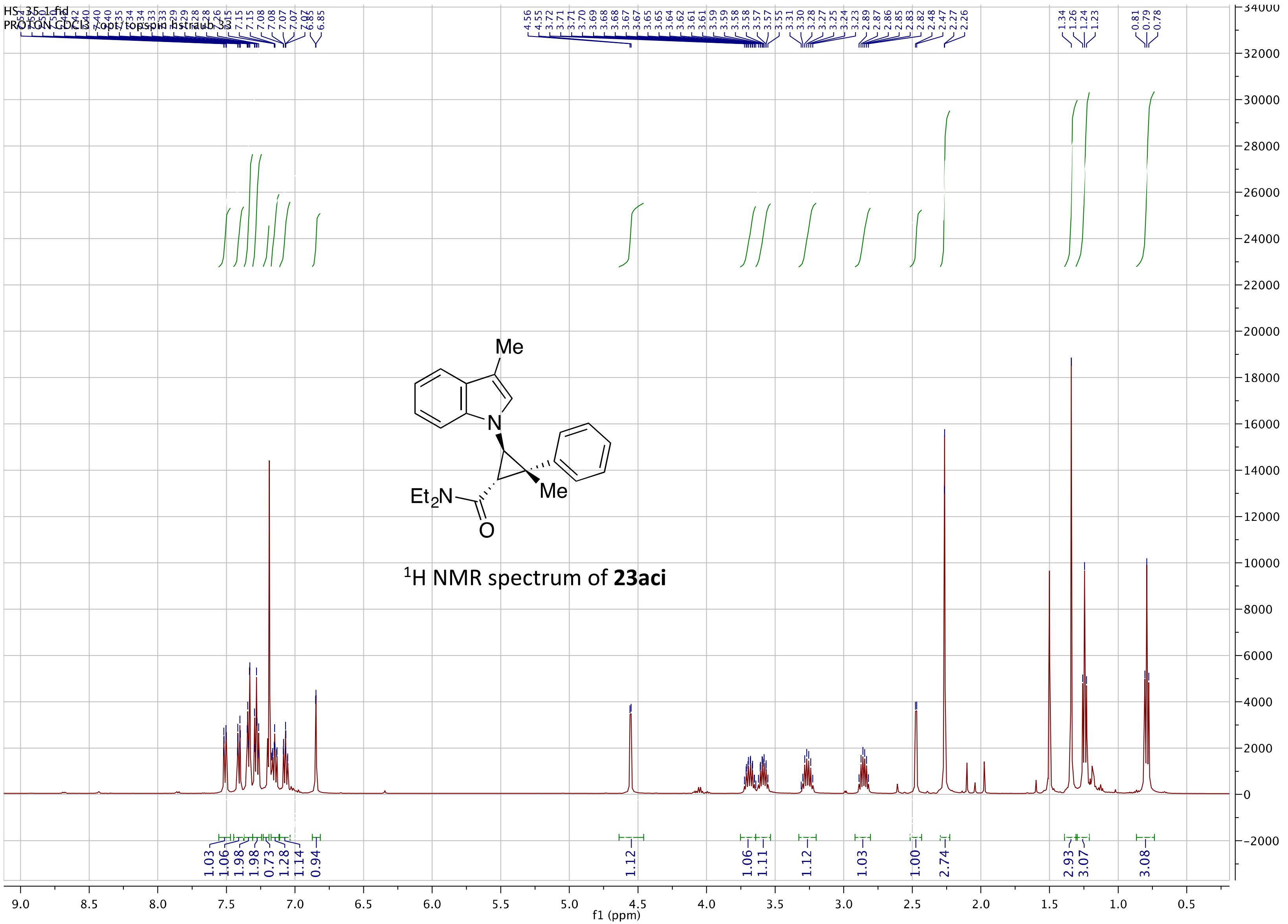

Figure S9. ^{1}H NMR spectrum of compound **23aci**

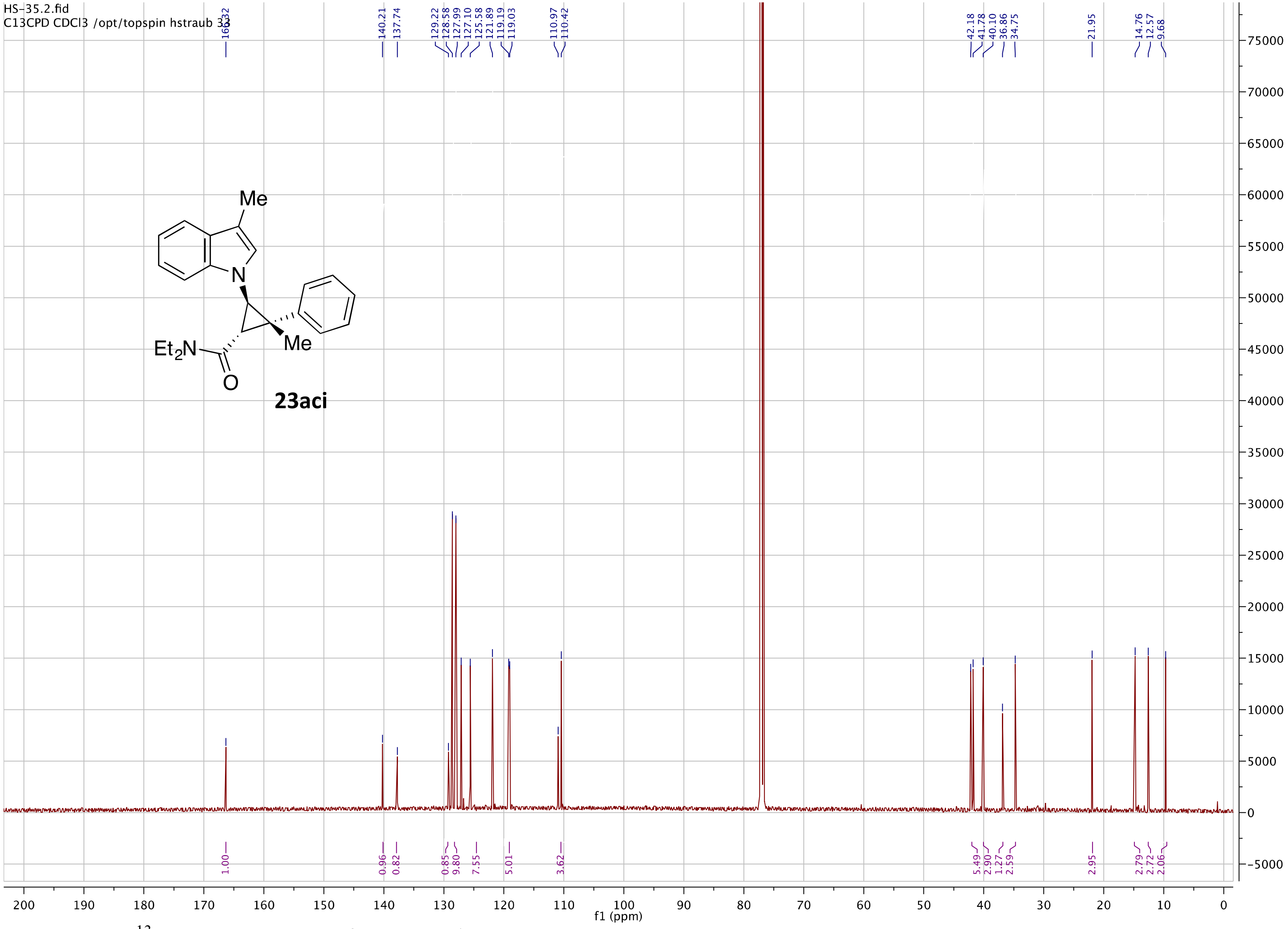

Figure S10. ^{13}C NMR spectrum of compound **23aci**

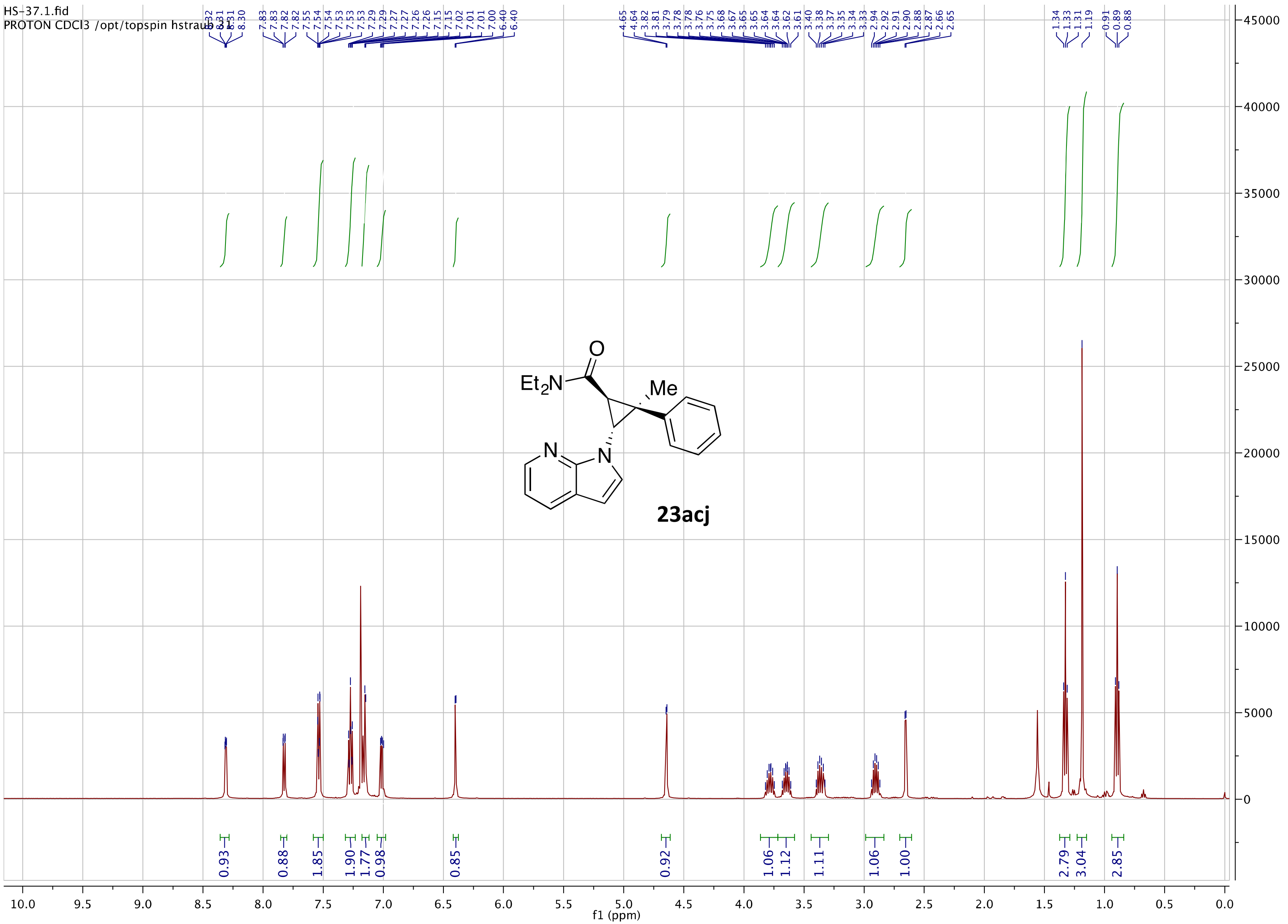

Figure S11. ^{1}H NMR spectrum of compound **23acj**

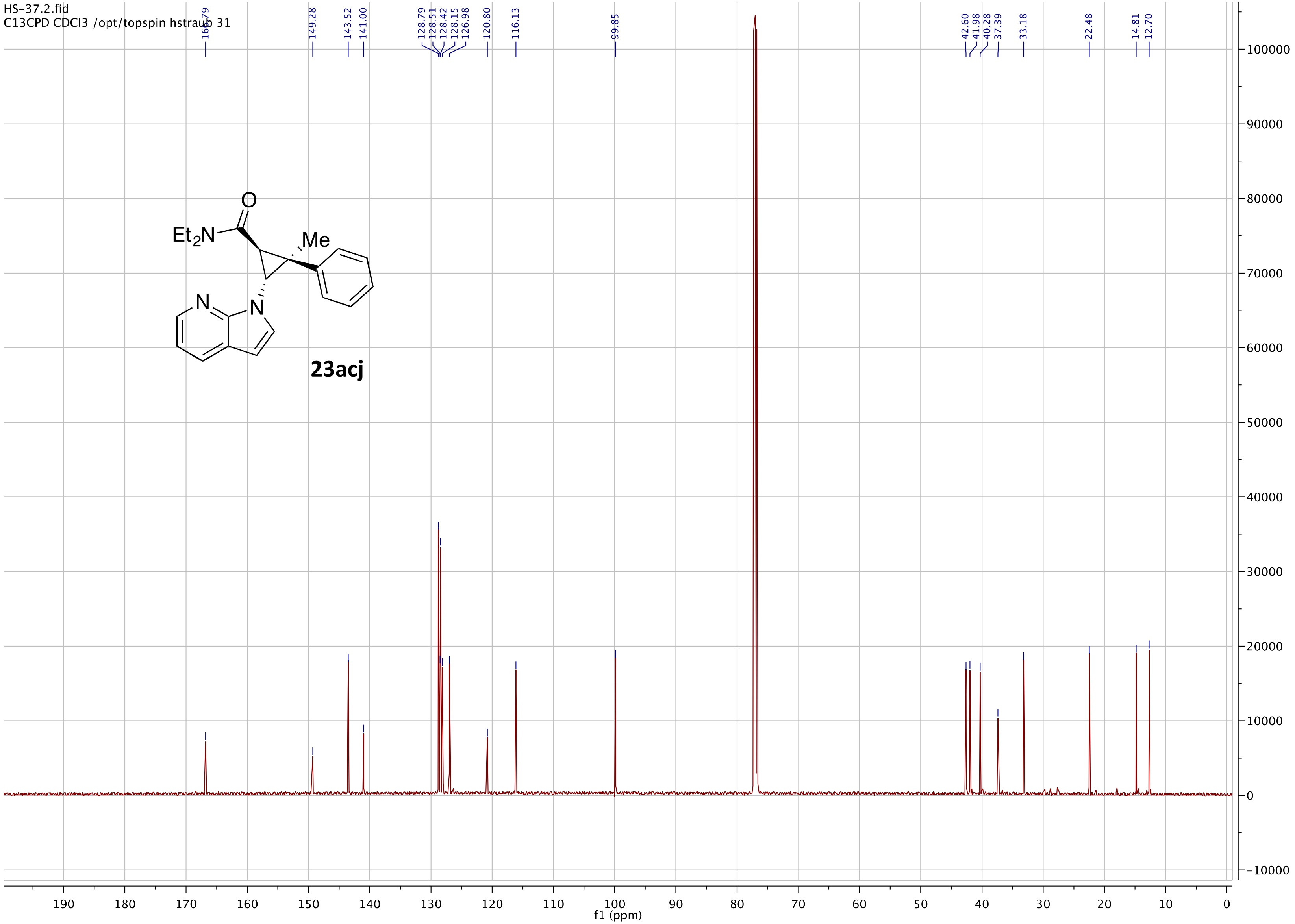

Figure S12. ^{13}C NMR spectrum of compound **23acj**

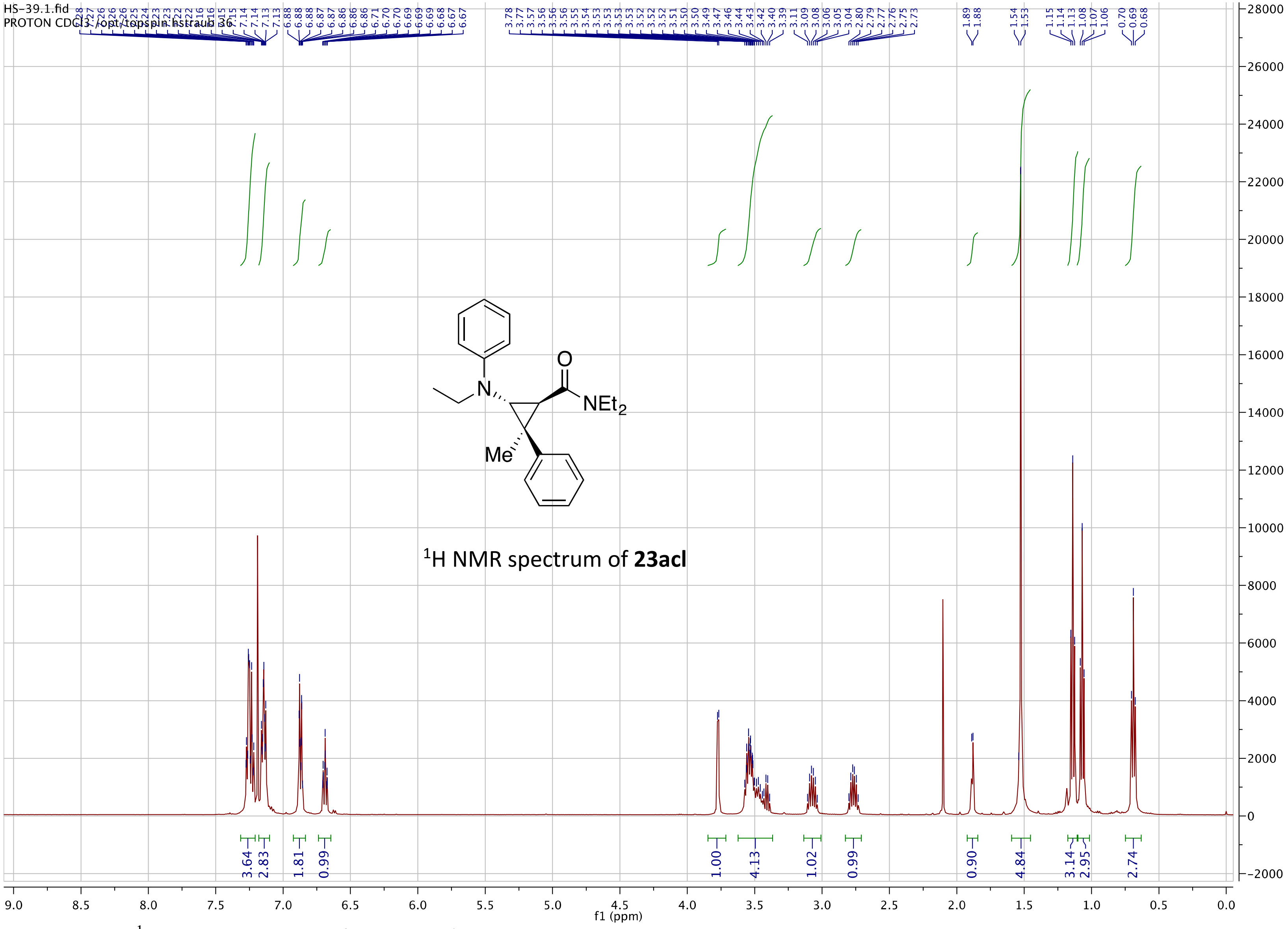

Figure S13. ^{1}H NMR spectrum of compound **23acl**

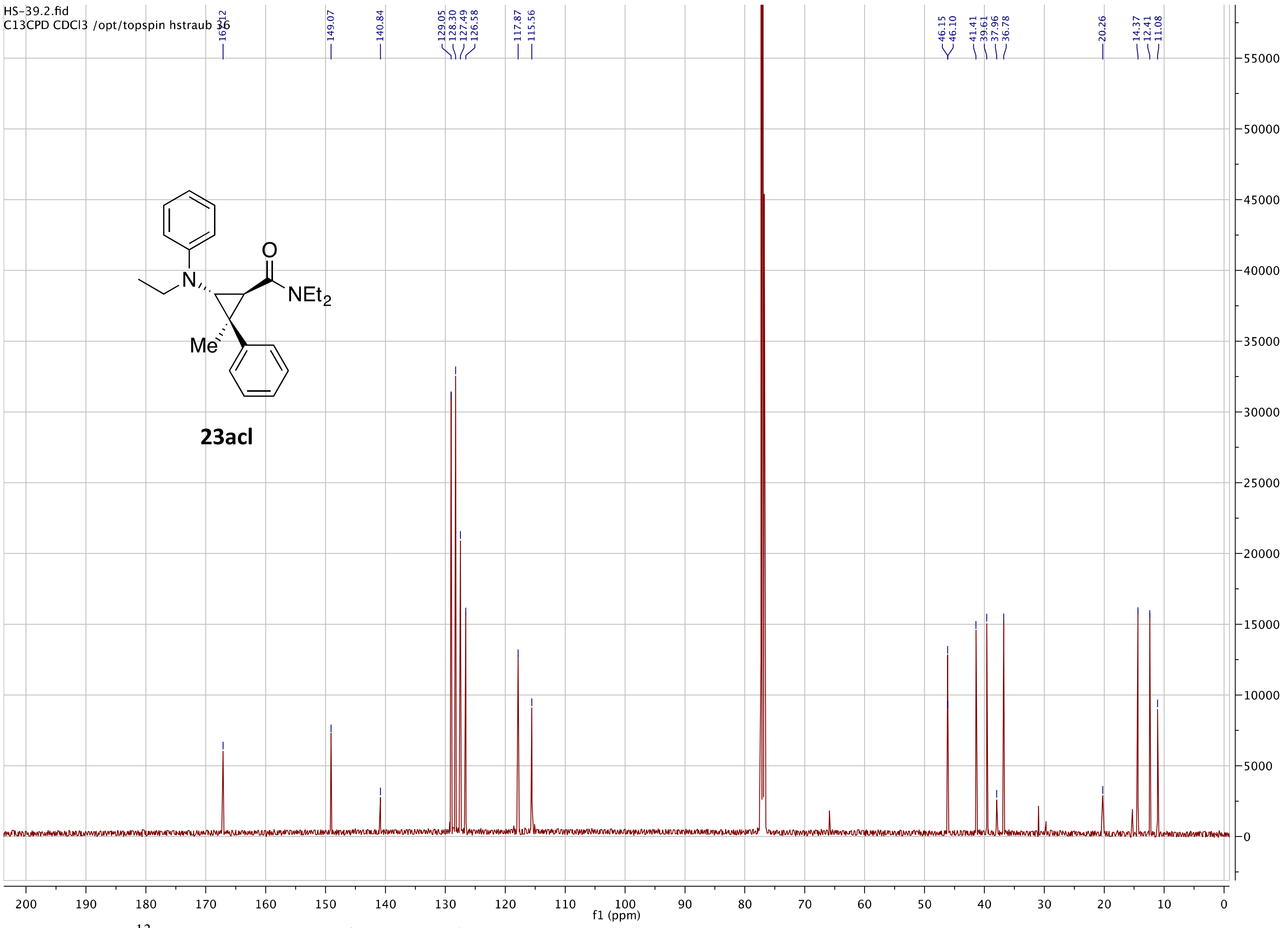

Figure S14. ^{13}C NMR spectrum of compound **23acl**

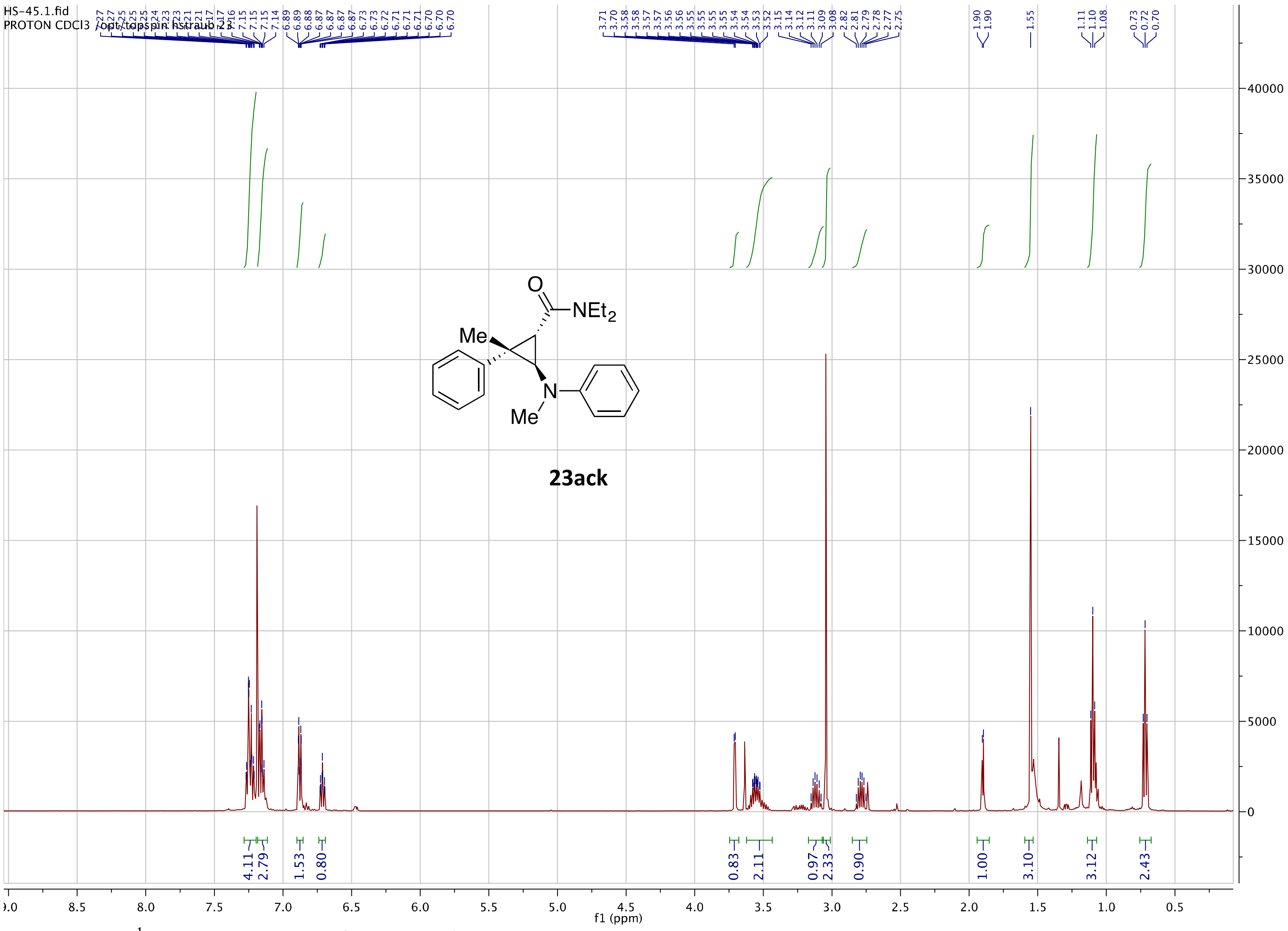

Figure S15. ^{1}H NMR spectrum of compound **23ack**

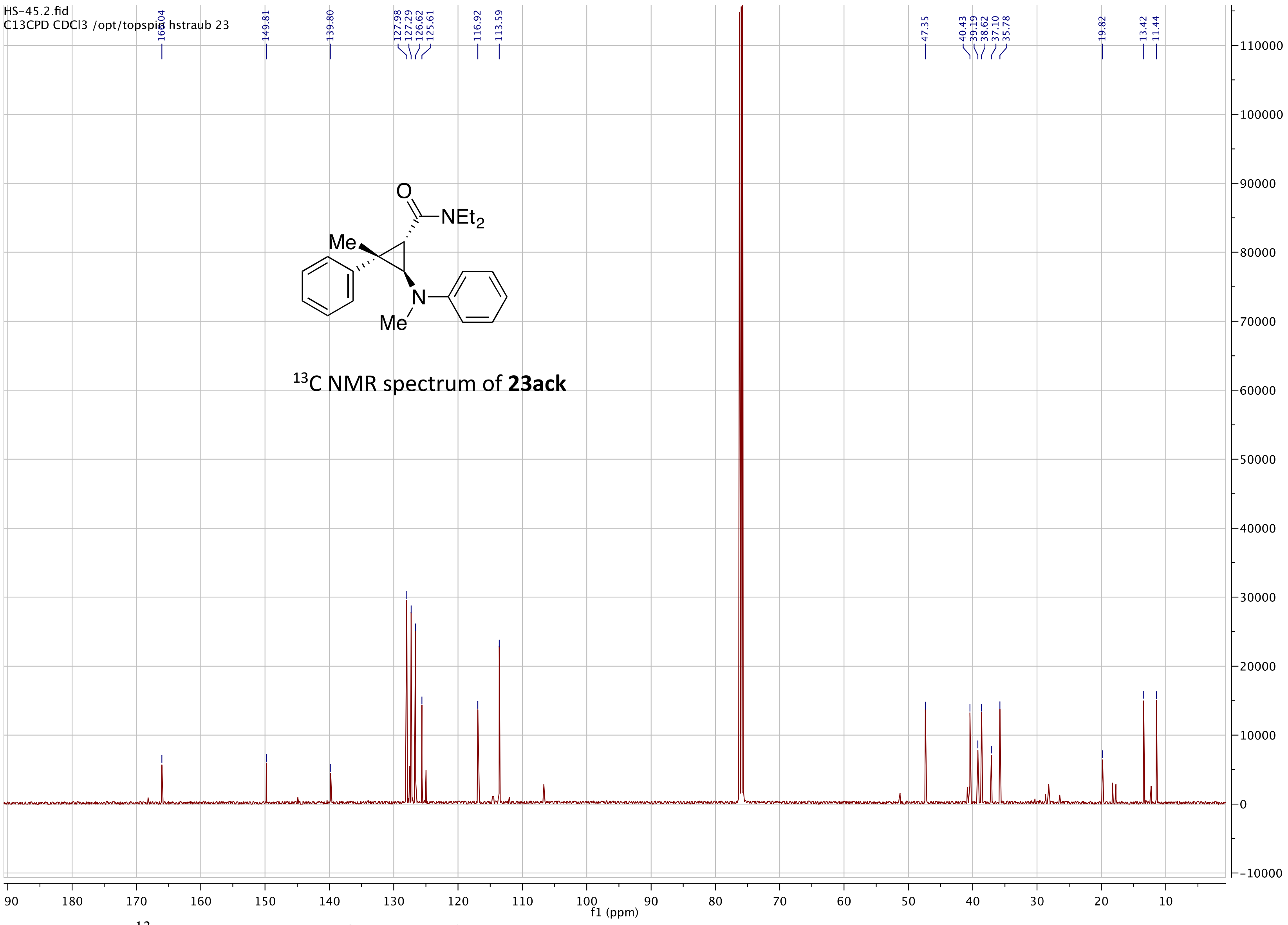

Figure S16. ^{13}C NMR spectrum of compound **23ack**

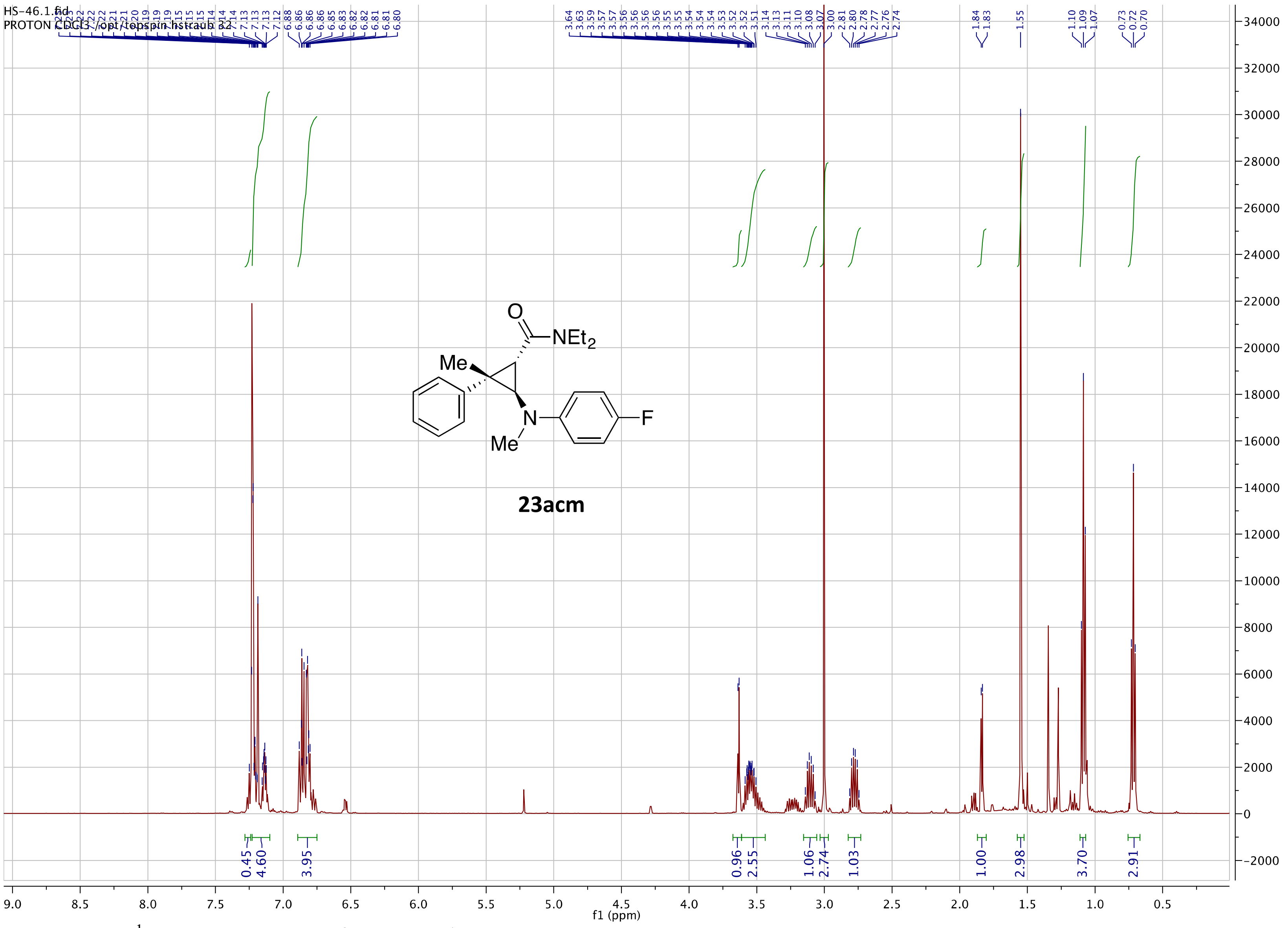

Figure S17. ^{1}H NMR spectrum of compound **23acm**

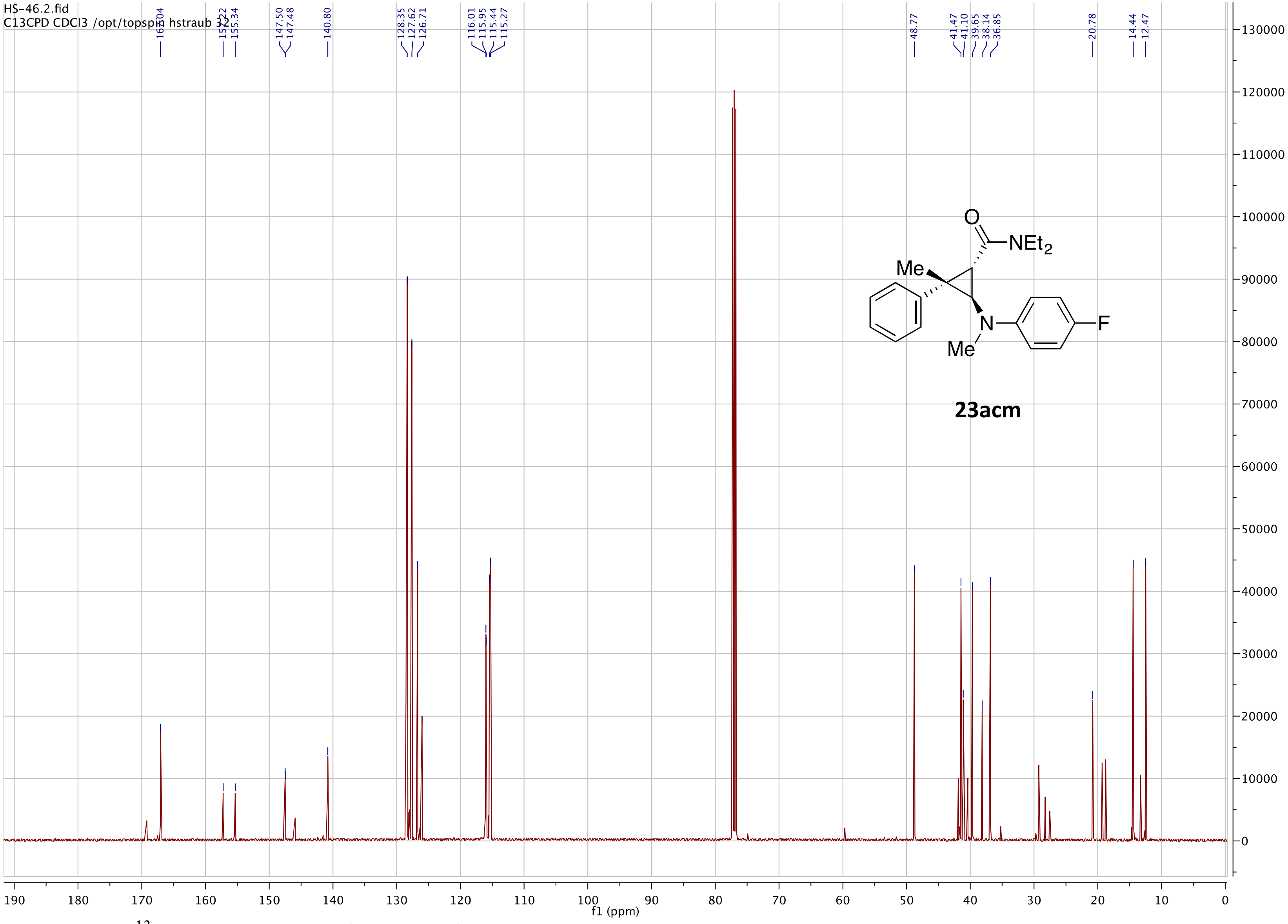

Figure S18. ^{13}C NMR spectrum of compound **23acm**

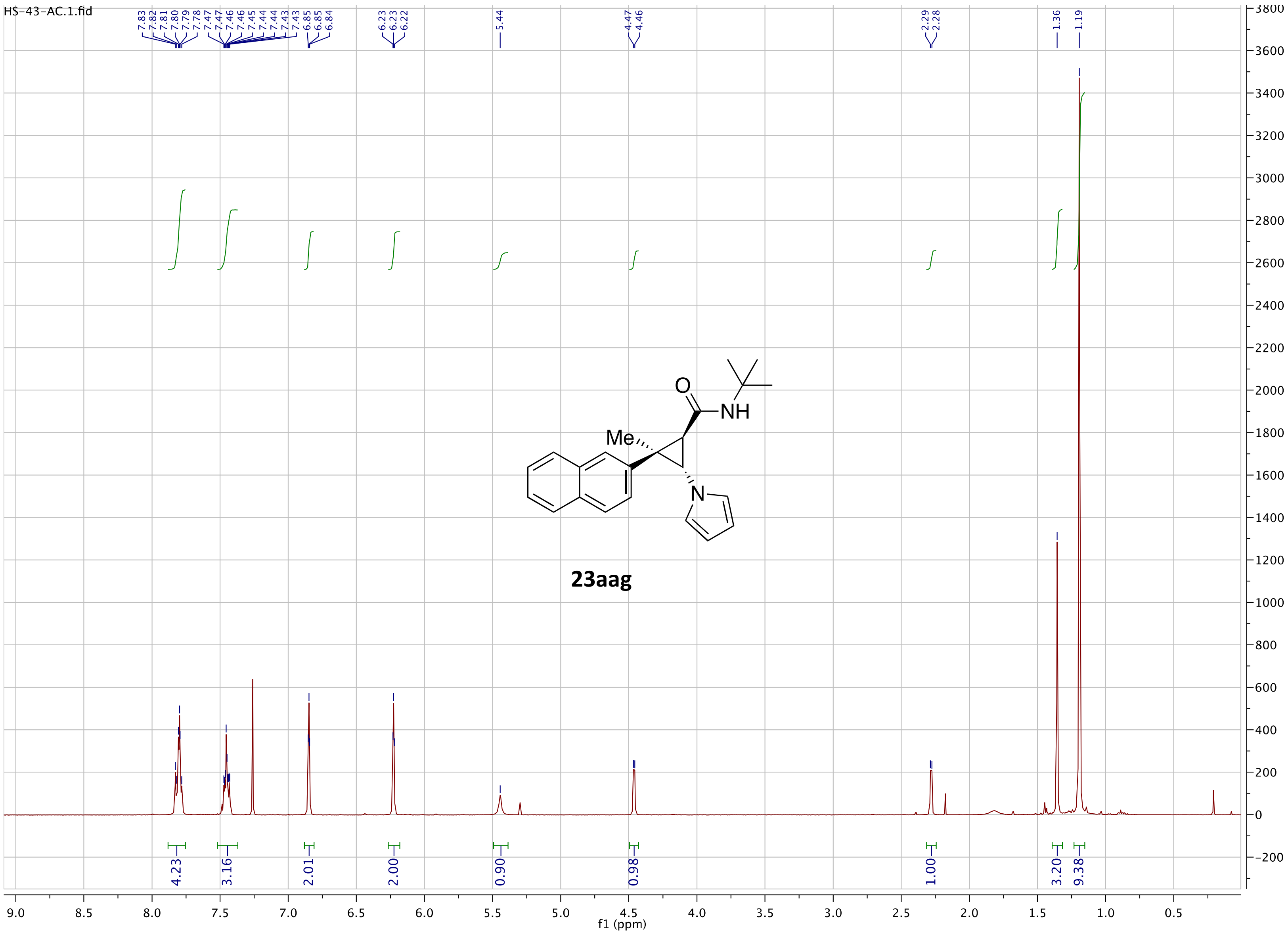

Figure S19. ^{1}H NMR spectrum of compound **23aag**

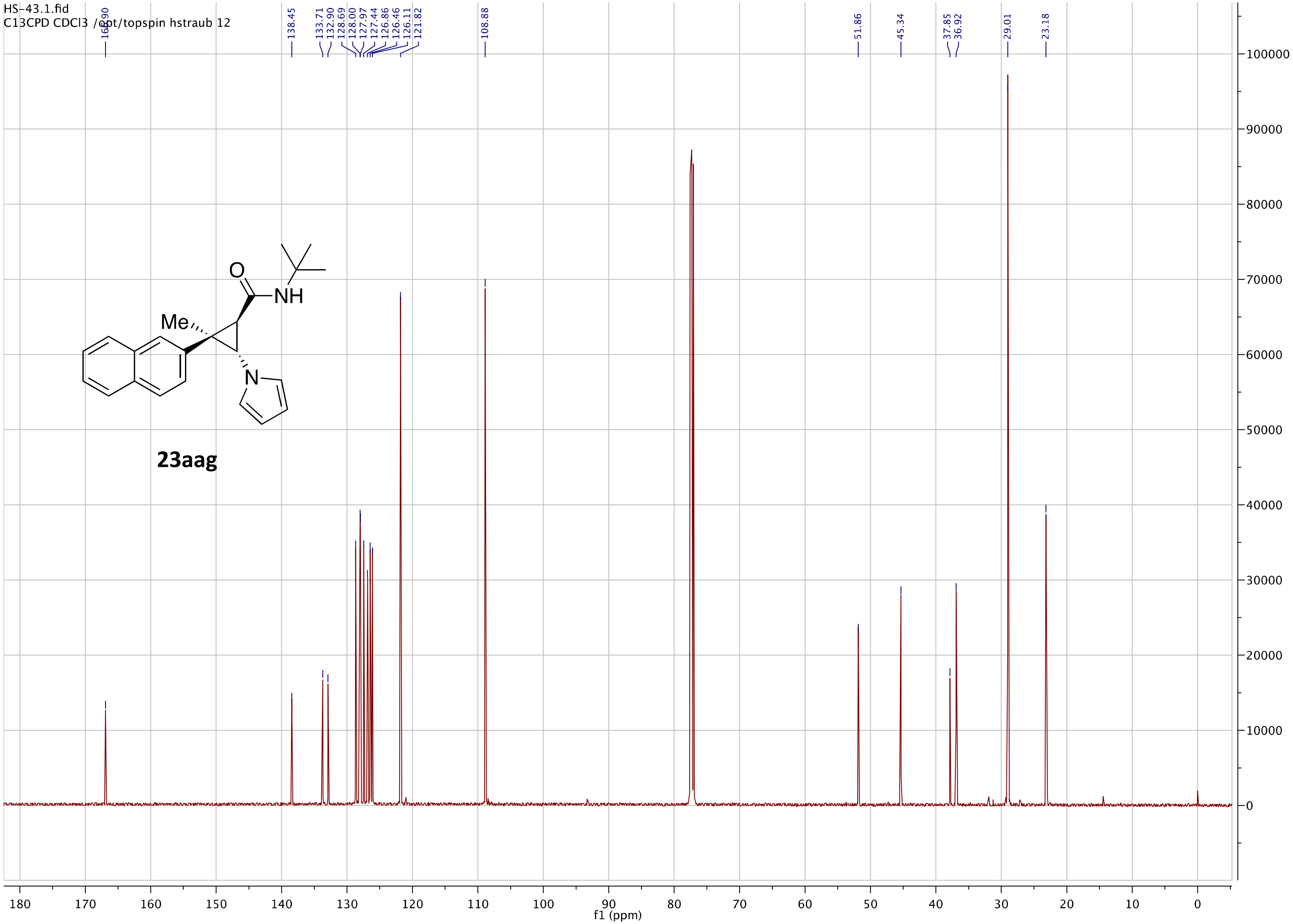

23aag

Figure S20. ^{13}C NMR spectrum of compound **23aag**

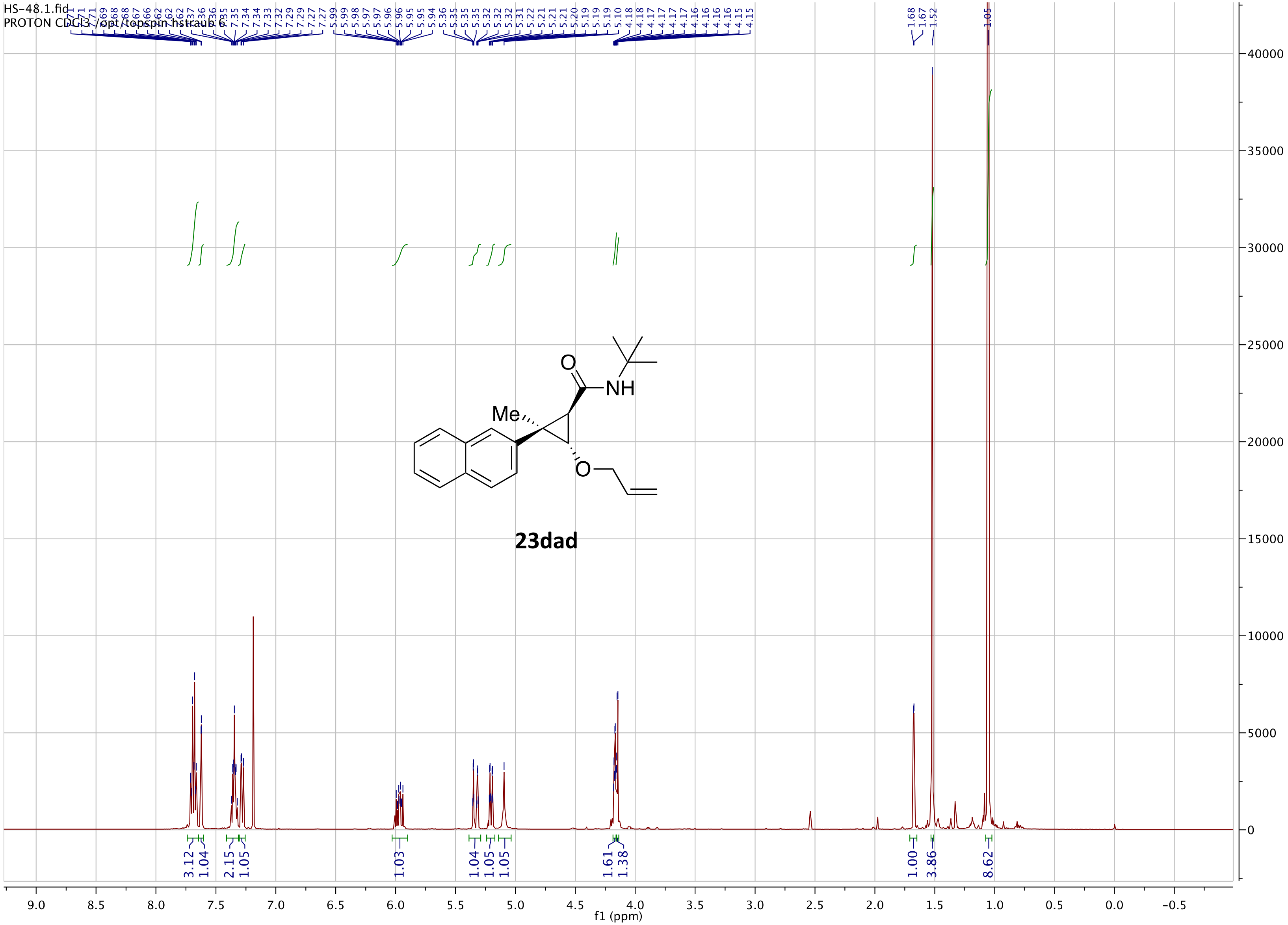

Figure S21. ^{1}H NMR spectrum of compound **23dad**

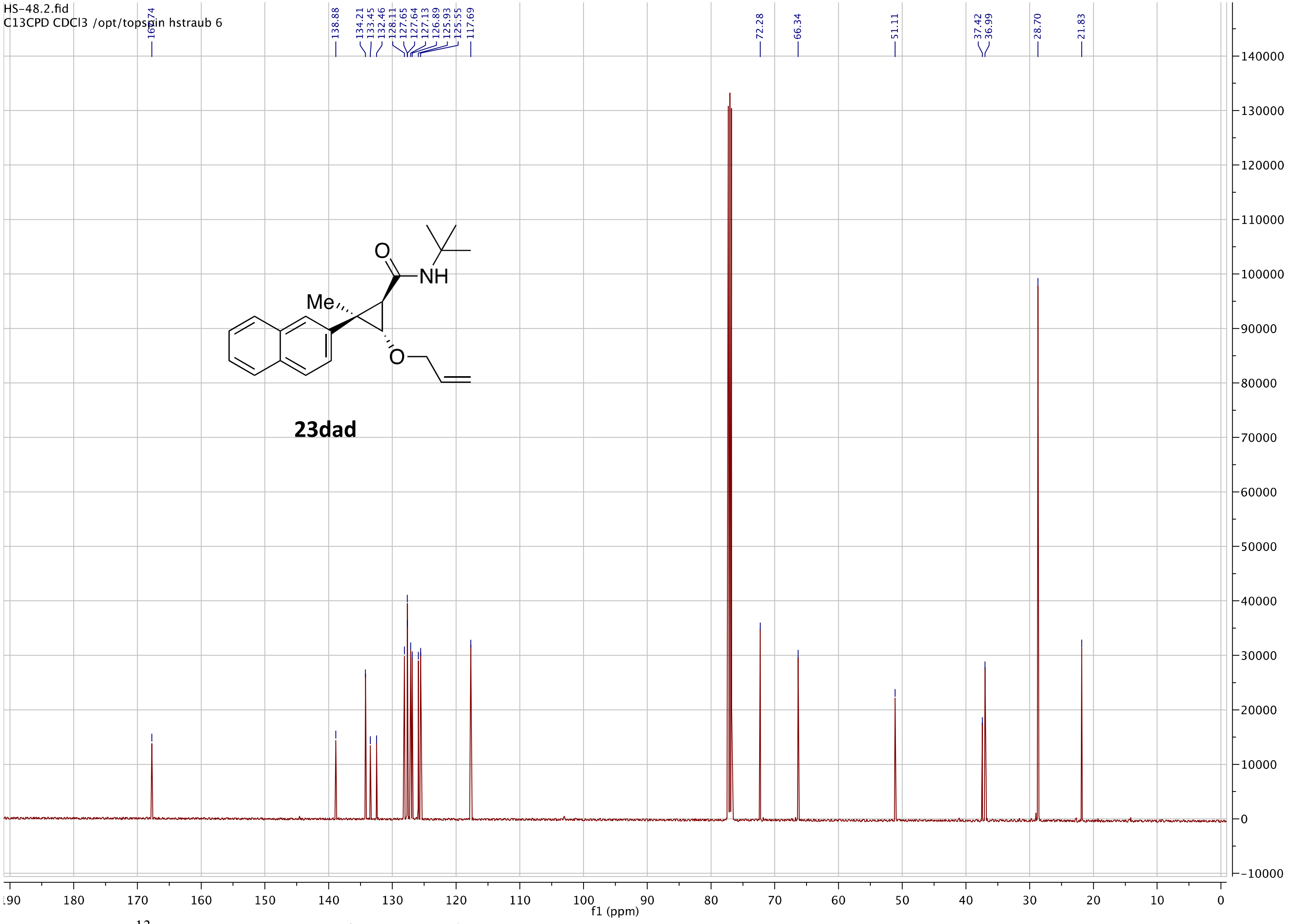

Figure S22. ^{13}C NMR spectrum of compound **23dad**

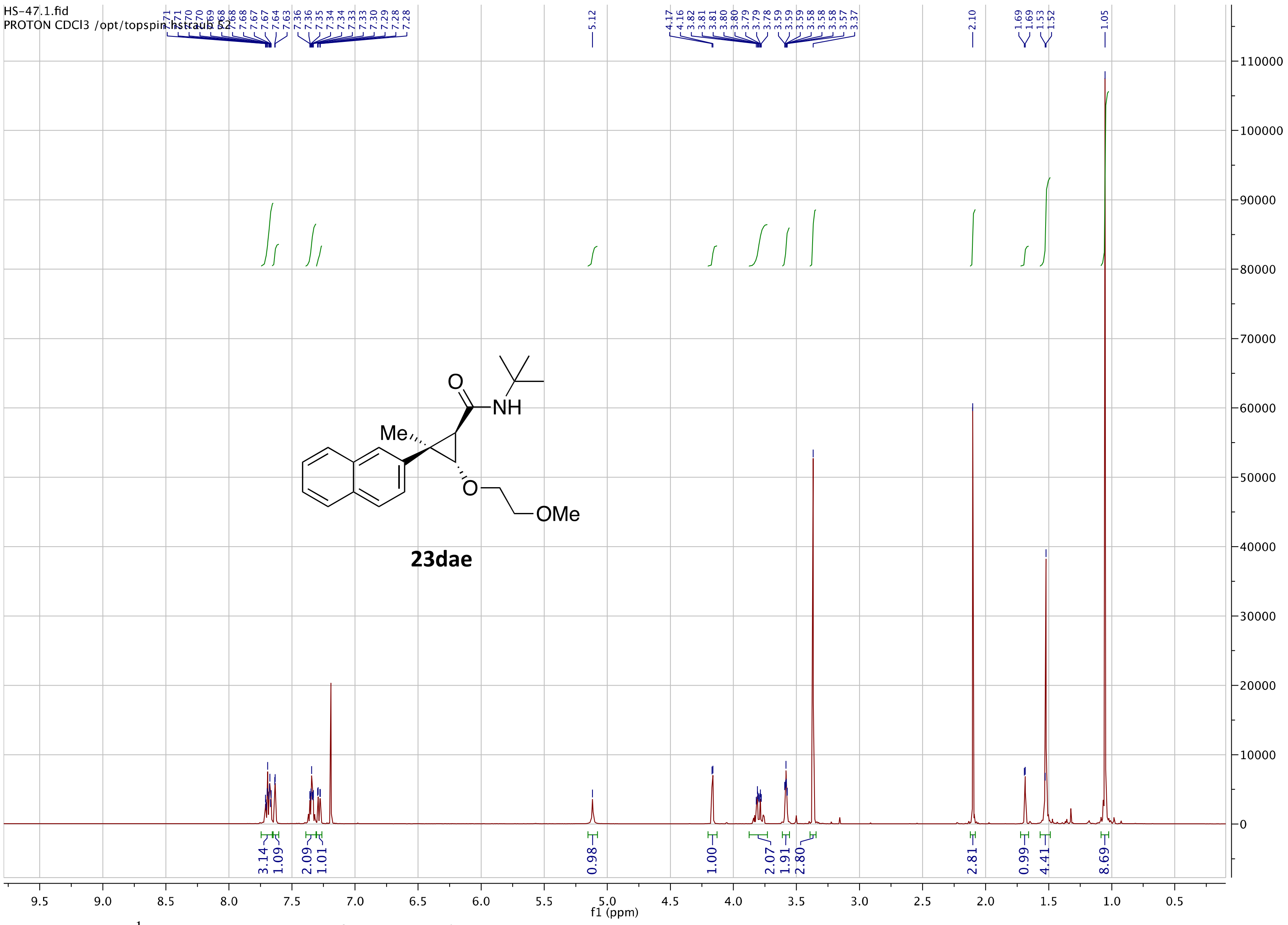

Figure S23. ^{1}H NMR spectrum of compound **23dae**

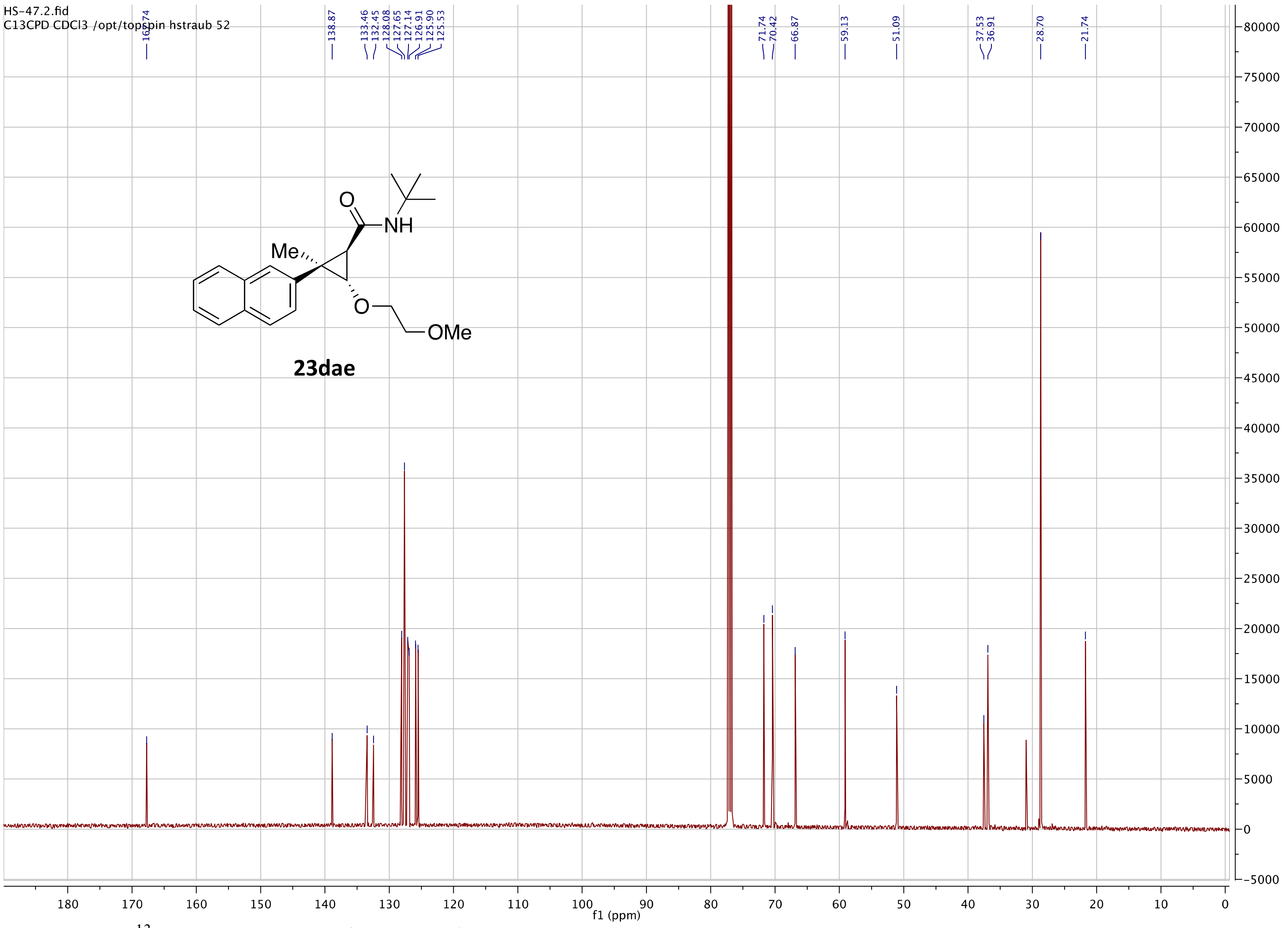

23dae

Figure S24. ^{13}C NMR spectrum of compound **23dae**

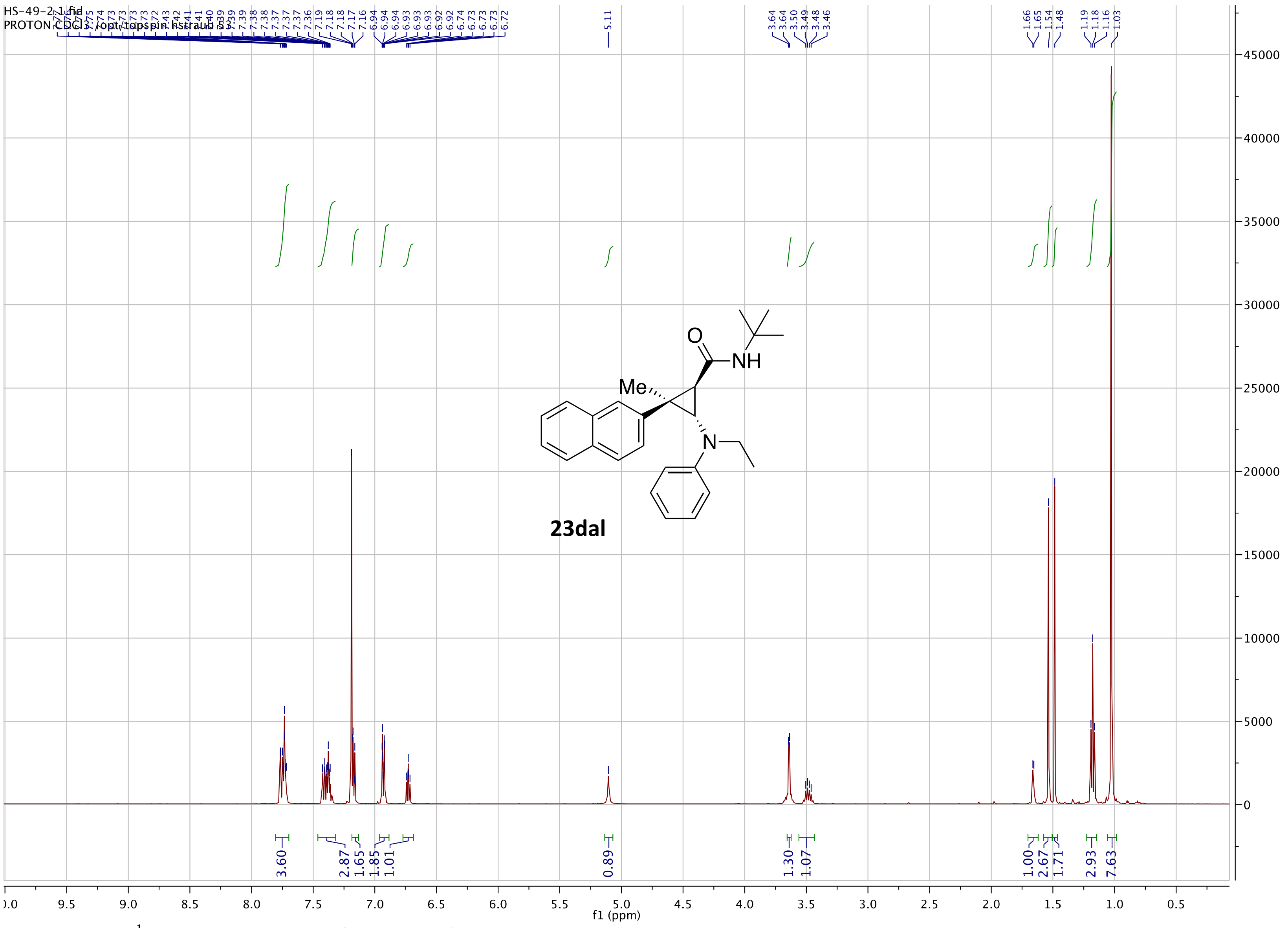

Figure S25. ^{1}H NMR spectrum of compound **23dal**

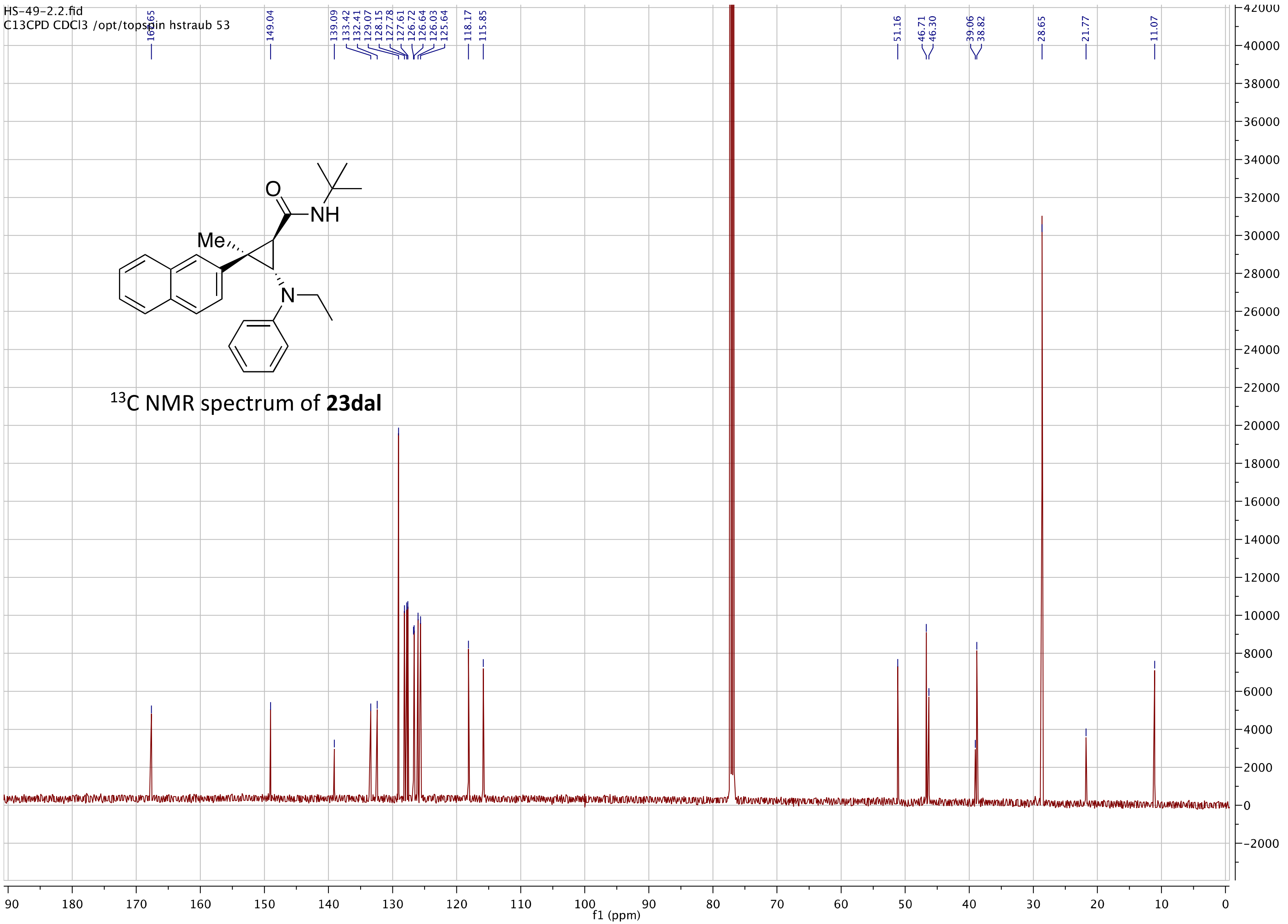

Figure S26. ^{13}C NMR spectrum of compound **23dal**

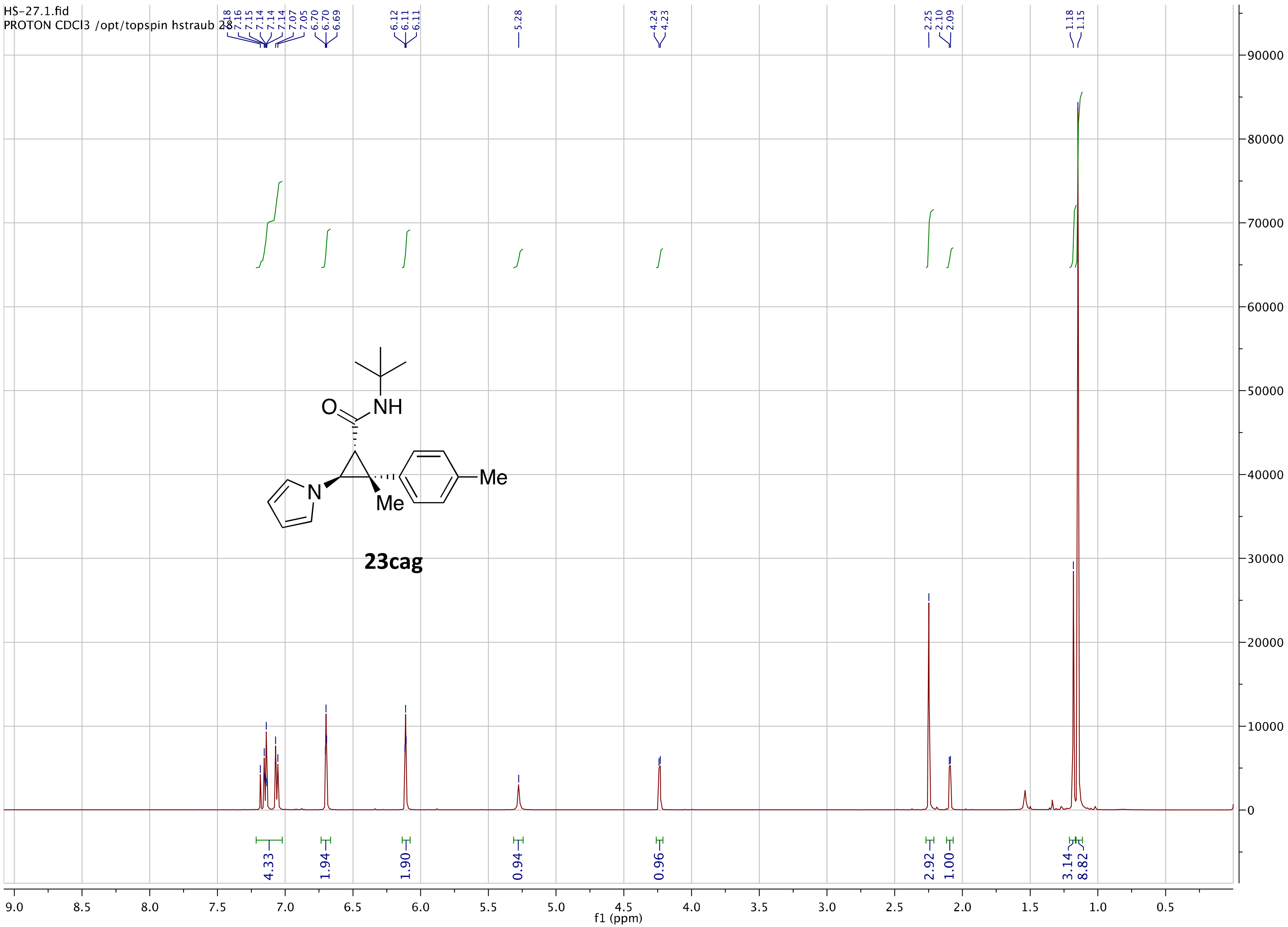

Figure S27. ^{1}H NMR spectrum of compound **23cag**

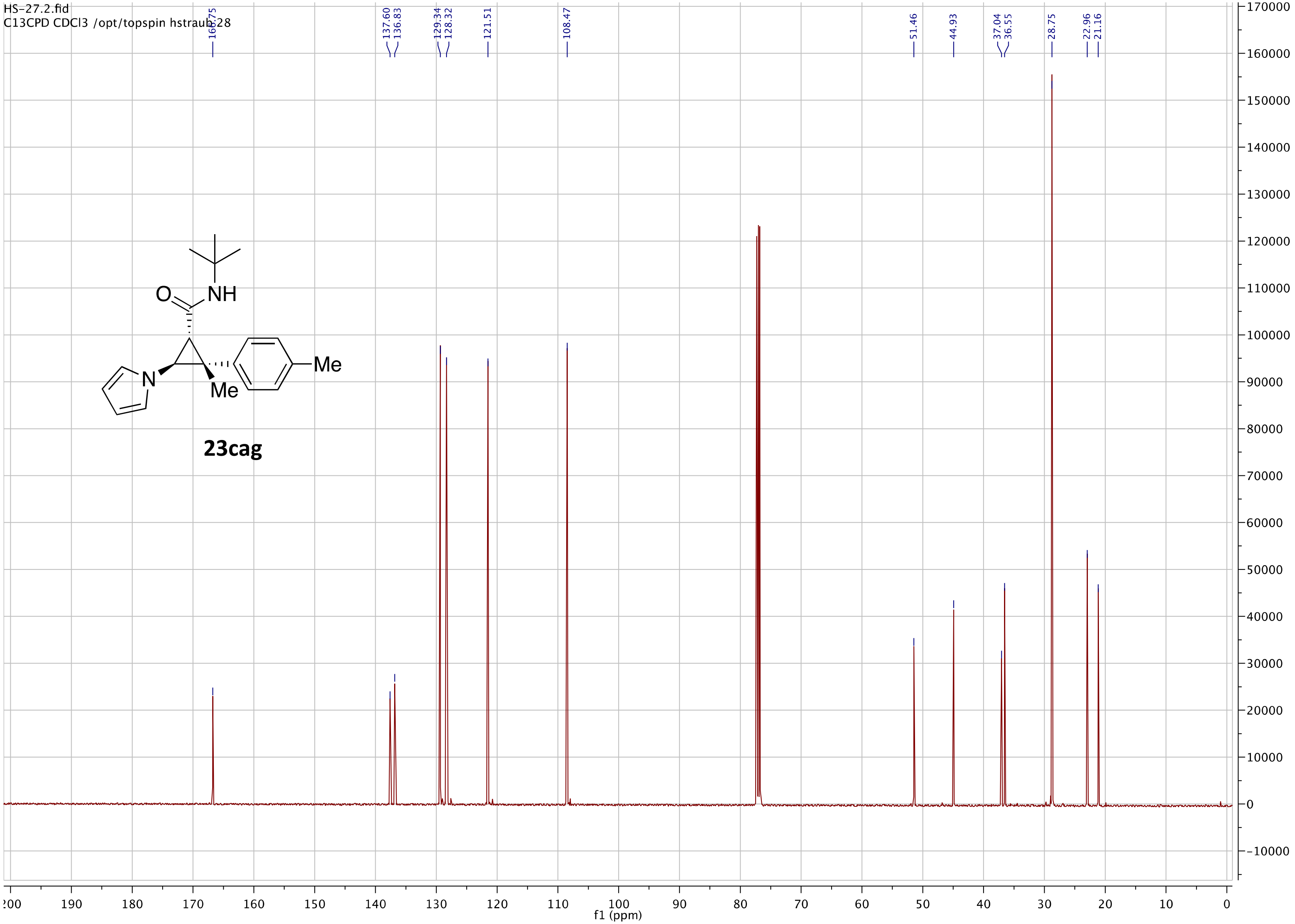

Figure S28. ^{13}C NMR spectrum of compound **23cag**

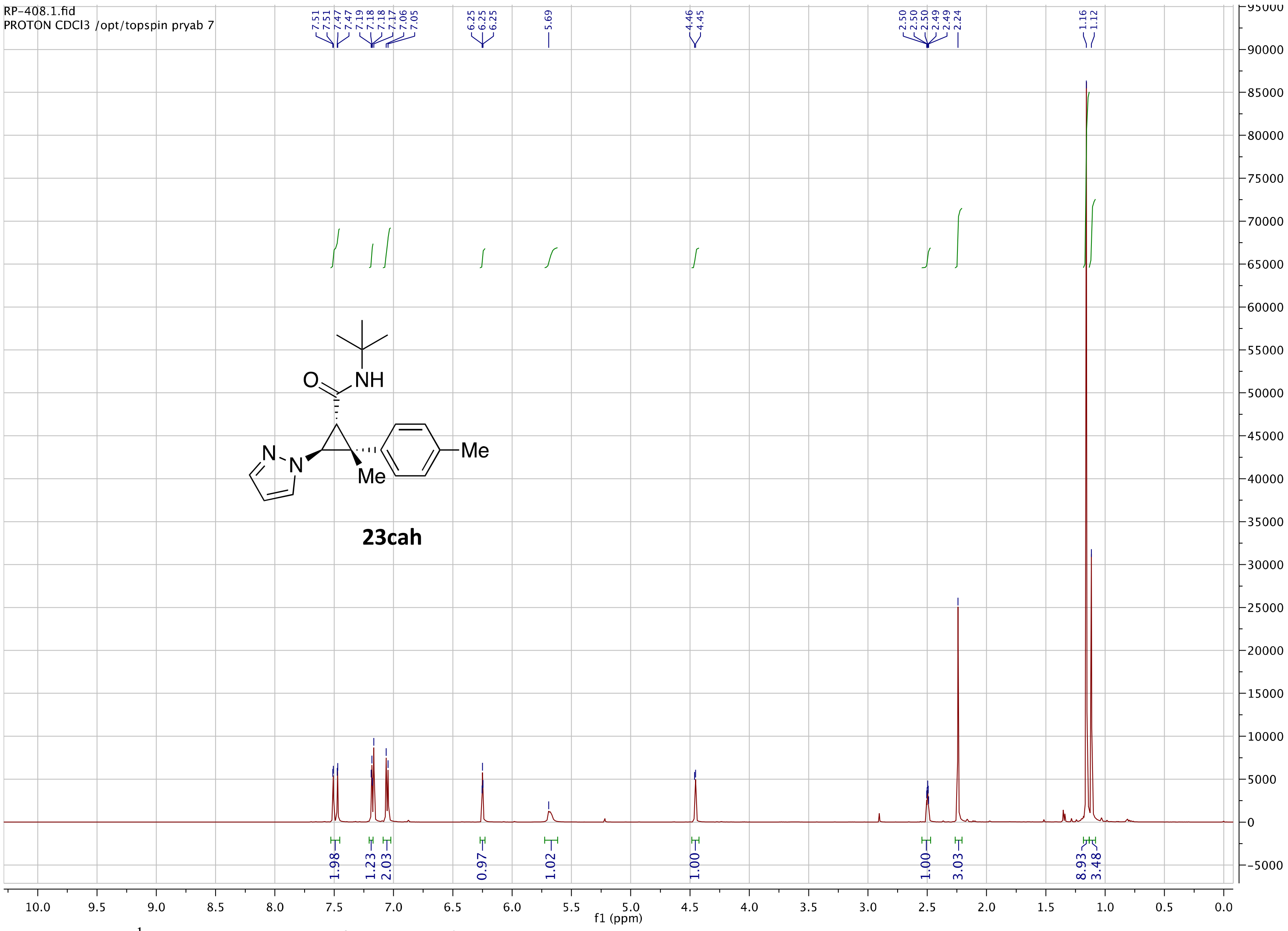

Figure S29. ^{1}H NMR spectrum of compound **23cah**

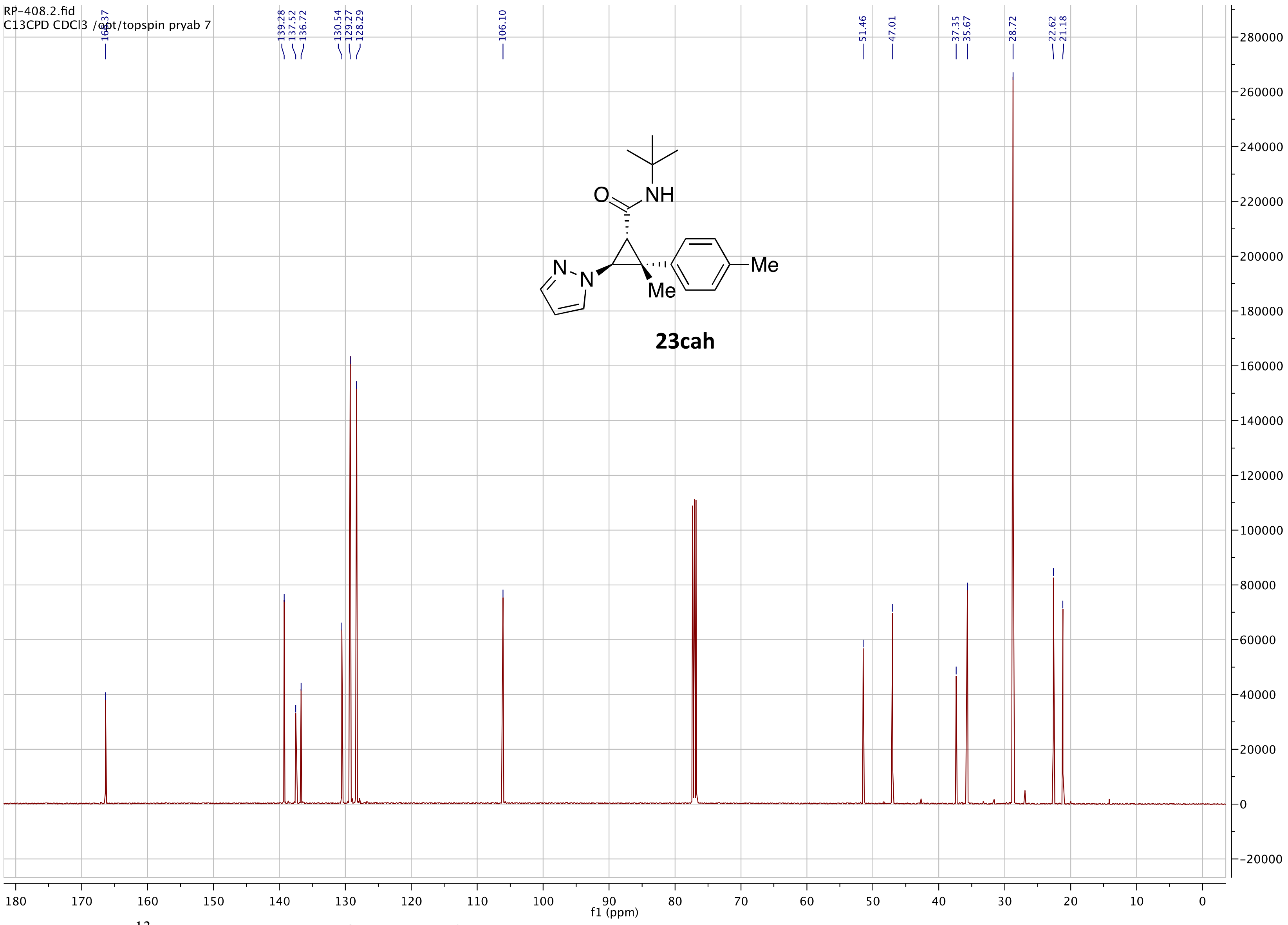

Figure S30. ^{13}C NMR spectrum of compound **23cah**

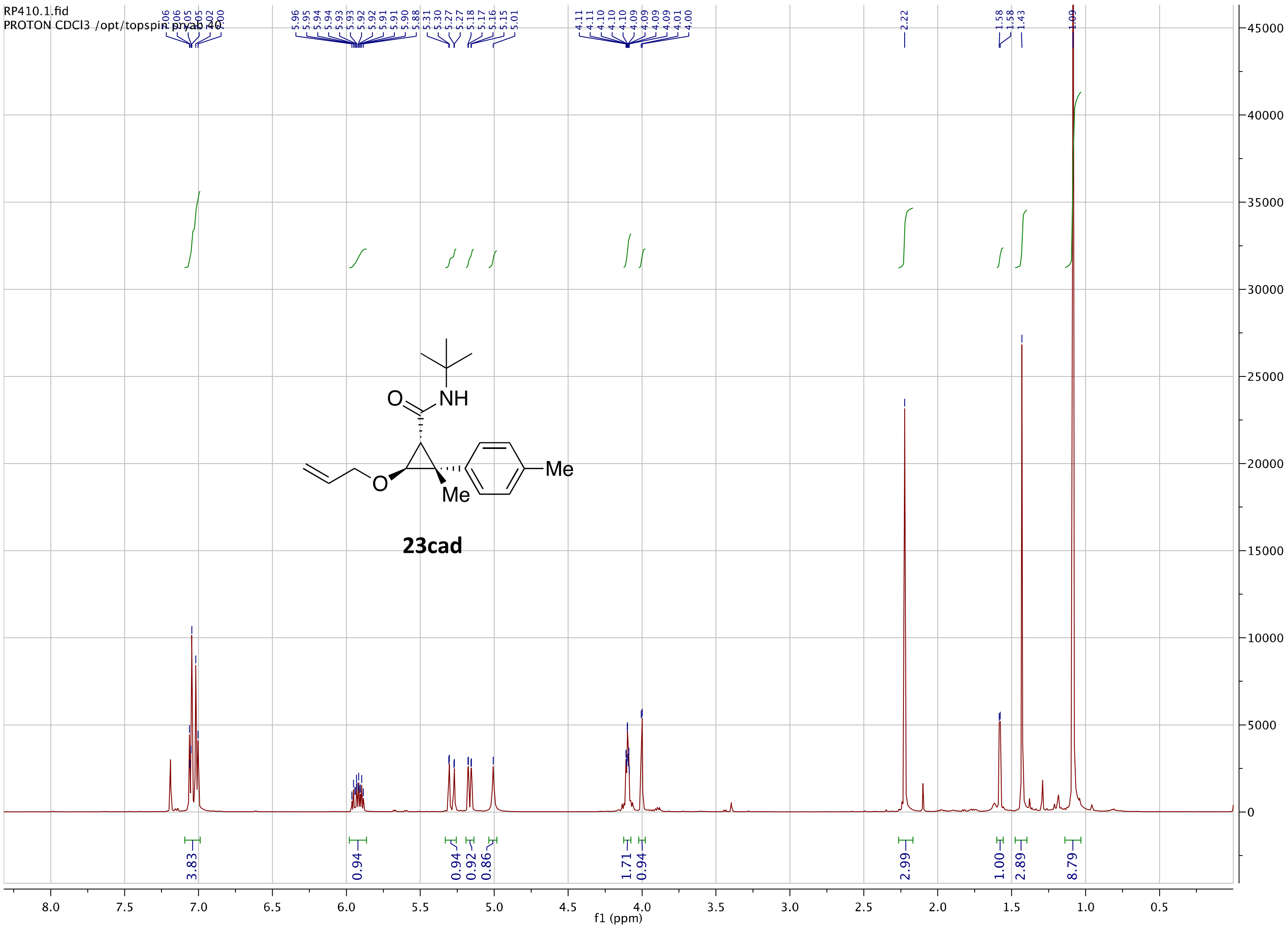

Figure S31. ^{1}H NMR spectrum of compound **23cad**

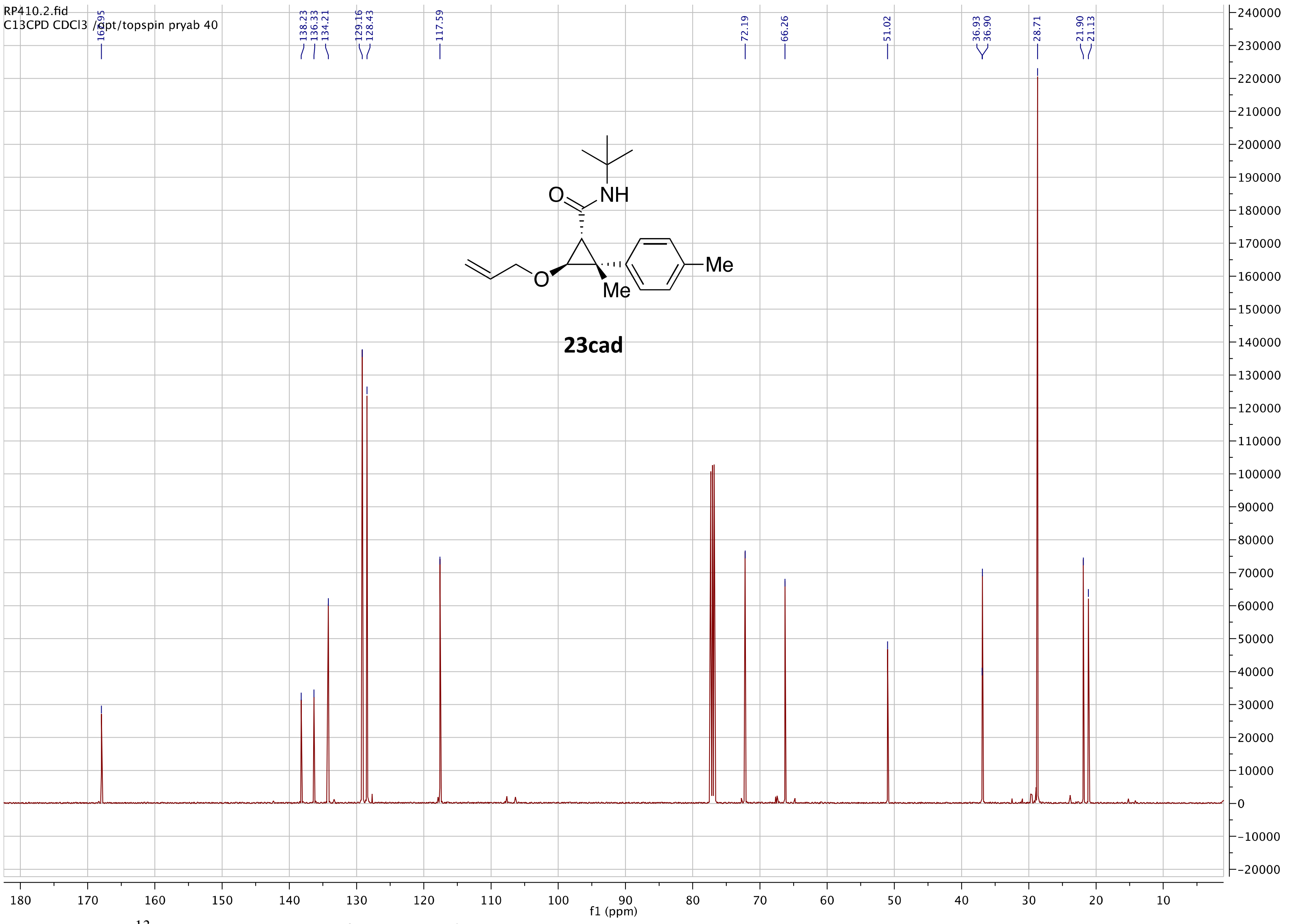

Figure S32. ^{13}C NMR spectrum of compound **23cad**
